# Genome-Wide Analysis Reveals the Potential Role of MYB Transcription Factors in Floral Scent Formation in *Hedychium coronarium*

**DOI:** 10.3389/fpls.2021.623742

**Published:** 2021-02-26

**Authors:** Farhat Abbas, Yanguo Ke, Yiwei Zhou, Yunyi Yu, Muhammad Waseem, Umair Ashraf, Chutian Wang, Xiaoyu Wang, Xinyue Li, Yuechong Yue, Rangcai Yu, Yanping Fan

**Affiliations:** ^1^The Research Center for Ornamental Plants, College of Forestry and Landscape Architecture, South China Agricultural University, Guangzhou, China; ^2^College of Life Sciences, South China Agricultural University, Guangzhou, China; ^3^College of Economics and Management, Kunming University, Kunming, China; ^4^College of Horticulture, South China Agricultural University, Guangzhou, China; ^5^Department of Botany, Division of Science and Technology, University of Education, Lahore, Punjab, Pakistan; ^6^Guangdong Key Laboratory for Innovative Development and Utilization of Forest Plant Germplasm, South China Agricultural University, Guangzhou, China

**Keywords:** *Hedychium coronarium*, MYB, terpenes, floral scent, structural genes

## Abstract

The MYB gene family is one of the largest groups of transcription factors (TFs) playing diverse roles in several biological processes. *Hedychium coronarium* (white ginger lily) is a renowned ornamental plant both in tropical and subtropical regions due to its flower shape and strong floral scent mainly composed of terpenes and benzenoids. However, there is no information available regarding the role of the MYB gene family in *H. coronarium*. In the current study, the MYB gene family was identified and extensively analyzed. The identified 253 *HcMYB* genes were unevenly mapped on 17 chromosomes at a different density. Promoter sequence analysis showed numerous phytohormones related to *cis*-regulatory elements. The majority of *HcMYB* genes contain two to three introns and motif composition analysis showed their functional conservation. Phylogenetic analysis revealed that HcMYBs could be classified into 15 distinct clades, and the segmental duplication events played an essential role in the expansion of the *HcMYB* gene family. Tissue-specific expression patterns of *HcMYB* genes displayed spatial and temporal expression. Furthermore, seven *HcMYB* (*HcMYB7/8/75/79/145/238/248*) were selected for further investigation. Through RT-qPCR, the response of candidates *HcMYB* genes toward jasmonic acid methyl ester (MeJA), abscisic acid (ABA), ethylene, and auxin was examined. Yeast one-hybrid (Y1H) assays revealed that candidate genes directly bind to the promoter of bottom structural volatile synthesis genes (*HcTPS1*, *HcTPS3*, *HcTPS10*, and *HcBSMT2*). Moreover, yeast two-hybrid (Y2H) assay showed that HcMYB7/8/75/145/248 interact with HcJAZ1 protein. In *HcMYB7/8/79/145/248*-silenced flowers, the floral volatile contents were decreased and downregulated the expression of key structural genes, suggesting that these genes might play crucial roles in floral scent formation in *H. coronarium* by regulating the expression of floral scent biosynthesis genes. Collectively, these findings indicate that *HcMYB* genes might be involved in the regulatory mechanism of terpenoids and benzenoid biosynthesis in *H. coronarium*.

## Introduction

Transcription factors (TFs) are proteins typically comprising of two functional domains involved in DNA-binding and transcriptional activation or repression of gene expression in response to internal or external stimuli (Riechmann et al., 2000; Amoutzias et al., 2007). Identification and deciphering the molecular function of TFs in regulating the gene expression provide insight into the signal transduction pathways and stress responses in different crops. TFs can be divided into different families based on DNA-binding domains that regulate the specific target genes. Among different TFs families, MYB is one of the largest and functionally diverse TFs superfamily found in nearly all eukaryotes. They are involved in a variety of critical processes, such as controlling plant growth and development, metabolism, physiological activities, cell morphology, and responses to environmental stresses (Baumann et al., 2007; Jung et al., 2008; Rawat et al., 2009; Dubos et al., 2010; Cao et al., 2020). MYB gene was first identified in avian myeloblastosis virus (AMV) followed by their identification in slime mold, fungi, animals, and plants (Klempnauer et al., 1982; Paz-Ares et al., 1987; Rosinski and Atchley, 1998; Stracke et al., 2001). COLORED1 (C1) was the first functionally characterized MYB gene from *Zea mays* in the plant kingdom and was associated with the regulation of anthocyanin biosynthesis (Paz-Ares et al., 1987). Although MYB genes have been extensively studied (Du et al., 2009; Dubos et al., 2010), the role of several MYB TFs remains ambiguous.

MYB proteins contain conserved MYB DNA-binding domains approximately ∼50–53 amino acids in length at the N-terminus and three α-helices tryptophan residues capable of forming a helix-turn-helix (HLH) to stabilize DNA-binding domain. In contrast, the C-terminal has diverse activation domains that play pivotal regulatory roles in these TFs (Ogata et al., 1996). Based on the number of repeats, MYB TFs are classified into four different groups: 1R, R2R3, 3R, and 4R-MYB (Stracke et al., 2001; Dubos et al., 2010). Unlike in animals, R2R3-MYB domain proteins are prevalent in plants (Martin and Paz-Ares, 1997; Dubos et al., 2010). The evolution of R2R3-MYB genes from R1R2R3-MYB genes by the loss of R1 repeat or duplication of R1 repeat from the 1R-MYB genes following expansion in plants has been proposed (Rosinski and Atchley, 1998; Jiang et al., 2004).

MYB proteins have been reported to be involved in controlling or regulating a wide range of processes, such as growth and development, flavonoid/phenylpropanoid metabolism, anthocyanin biosynthetic pathway, sugar signaling, secondary wall biosynthesis, and resistance to biotic and abiotic stresses (McCarthy et al., 2010; Medina-Puche et al., 2014; Tuan et al., 2015; Chen Y.-S. et al., 2017). In *Arabidopsis thaliana*, the overexpression of *AtMYB96* increases drought tolerance via coordinating auxin and ABA signaling pathway as well as by regulating the Lipid transfer protein 3 (*LTP3*) target gene (Seo et al., 2009). *AtMYB44* and *AtMYB60* were involved in drought stress by regulating the stomatal movement (Jung et al., 2008). *Gossypium barbadense MYB5* promotes drought tolerance in transgenic tobacco and cotton plants (Chen et al., 2015). Several *MYB* genes play roles in induced resistance toward insect herbivores or against mechanical wounding, such as *AtMYB15*, *AtMYB34*, *AtMYB51*, and *AtMYB75*. However, rice *OsLTR1* regulates jasmonic acid (JA)-dependent defense responses (Johnson and Dowd, 2004; Liu et al., 2010). Jasmonate ZIM domain protein (JAZ) is a key component of the JA signaling pathway and plays a crucial role in plant responses to environmental stimuli. The suppressor proteins JAZ is the main component in the crosstalk as it interacts with other hormone signaling pathways, such as auxin, ABA, salicylic acid (SA), and ethylene (Yang et al., 2019a). JAZ proteins interact with the other TFs, such as MYB/MYC/bHLH/WRKY/AP2/ERF/NAC to suppress the expression of jasmonate-responsive genes. Through the interaction between the MYB and JAZ, this process governs plant growth and development, abiotic stresses, defense resistance, and regulates various secondary metabolites (Zhou and Memelink, 2016). Moreover, *AtMYB125* regulates male germ cell division and differentiation (Brownfield et al., 2009), while *AtMYB33* and *AtMYB65* facilitate carpel development and ensure the production of viable pollen in anther (Millar and Gubler, 2005). *AtMYB115*/*AtMYB118* plays an essential role in embryogenesis (Wang et al., 2009). Likewise, *AtMYBL2* acts as a transcriptional repressor and inhibits the accumulation of proanthocyanin content in *Arabidopsis* (Dubos et al., 2008). Meanwhile, the grape *VvMYB4*-like gene and the soybean *GmMYB100* negatively regulate flavonoid biosynthesis in plants (Yan et al., 2015; Pérez-Díaz et al., 2016). Similarly, *R2R3-MybA* TFs from *Muscari armeniacum* was involved in the biosynthesis of anthocyanin and can be used in flower color modification (Chen et al., 2019). Similarly, the characterization of novel litchi R2R3-MYB revealed its involvement in tissue acidification and anthocyanin biosynthesis (Lai et al., 2019). *AtMYBL2/4/7* plays a crucial role in the regulation of flavonoid biosynthesis in Arabidopsis (Jin et al., 2000; Dubos et al., 2008). In apple, *MdMYB3* regulates the biosynthesis of anthocyanin through activating the numerous flavonoid pathway genes and is involved in flower development (Vimolmangkang et al., 2013). *GmMYB176* plays a pivotal role in flavonoid biosynthesis in *Glycine max* (Yi et al., 2010). *ODORANT1* (*ODO1*), EMISSION OF BENZENOID II (EOBII), EOBI, and LATE ELONGATED HYPOCOTYL (LHY) regulate flower scent production via activating scent-related genes in petunia (*Petunia hybrida*) (Verdonk et al., 2005; Spitzer-Rimon et al., 2010, 2012; Fenske et al., 2015). In *Pinus pinaster*, *MYB8* and *ELONGATED HYPOCOTYL* (*HY5*) controls the formation of phenylalanine and its metabolic channeling for lignin biosynthesis (El-Azaz et al., 2020). The *MYB10* and *FaEOBII* from strawberry (*Fragaria* × *ananassa*) were involved in the regulation of eugenol (Medina-Puche et al., 2014, 2015). *PpMYB15* and *PpMYBF1* from *Prunus persica* had flower-specific expression and involved in the regulation of flavanol biosynthesis (Cao et al., 2018).

*H. coronarium* (white ginger lily or butterfly ginger) is a perennial ornamental flowering plant native to the Eastern Himalayas region and southern China. Floral fragrance is one of the main characteristics of ornamental plants, which improves their economic and aesthetic values (Dudareva et al., 2013; Muhlemann et al., 2014; Abbas et al., 2017). *H. coronarium* flower emits a strong scent, which is a complex mixture of volatiles terpenes including β-ocimene, 1,8-cineole, linalool, eucalyptol (monoterpenes), α-farnesene, β-caryophyllene (sesquiterpenes), and benzenoids (Fan et al., 2003, 2007; Báez et al., 2011; Yue et al., 2014, 2015; Ke et al., 2019). However, there is no information about the role of MYB TFs in *H. coronarium*.

Previously, we identified key structural genes involved in volatile biosynthetic pathways, such as benzoic/salicylic acid methyltransferase gene (BSMT) *HcBSMT2* and the terpene synthases (TPSs) *HcTPS1*, *HcTPS3*, and *HcTPS10*. *HcBSMT2*, *HcTPS1*, *HcTPS3*, and *HcTPS10* are among the key structural genes involved in the biosynthetic pathway of floral scent formation (Yue et al., 2015). Functional characterization of *HcTPS1* and *HcTPS3* revealed their role in the formation of monoterpenes eucalyptol and β-ocimene, respectively. *HcTPS10* performs a dual function by interacting with both GPP and FPP to generates ocimene and α-farnesene, respectively. Furthermore, *HcTPS1* and *HcTPS3* showed a positive correlation with the emission of monoterpenes, a similar trend was observed for *HcTPS1*0 with the emission of sesquiterpene α-farnesene. On the other hand, *HcBSMT2* was specifically expressed in flowers and was involved in methyl benzoate formation (Yue et al., 2015). However, the transcriptional regulation of these genes is not elucidated. Regulatory proteins are important as they control the expression of structural genes and many structural genes are efficiently targeted for crop improvement (Bovy et al., 2007). In the current study, *HcMYB* genes were comprehensively analyzed. Moreover, Seven R2R3-HcMYB genes were functionally characterized and their role toward floral scent emission has been elucidated. This research will provide insights and will assist the scientists to further elucidate the biological roles of *MYB* genes in *H. coronarium*.

## Experimental Procedure

### Plant Materials and Growth Conditions

*H. coronarium* was planted in the growth chamber under conditions: 26 ± 2°C and 13/11 h light/dark photoperiod. For tissue-specific expression analysis, three different plant parts; flowers at full bloom, green leaves, and rhizomes were used. For the flowers, different flower developmental stages including D1; squaring stage, D2; half-open stage, D3; full-bloom, D4; senescence stage were selected. For different hormone treatments, the flower stems were shortly cut into 40 cm and then placed in sterilized water containing 100 μM IAA, ABA (200 μM), and MeJA (100 μM). For ethylene treatment, flowers were incubated with 10 μl/L of ethylene for 12 h in a sealed bottle. After that, the flowers were placed in an artificial growth chamber with a 14/10 h day/light photoperiod at 25°C. The floral volatile compound analysis was performed at the full-bloom stage of the treated flowers and immediately frozen in liquid nitrogen and stored at −80°C. *Nicotiana benthamiana* and *Arabidopsis thaliana* plants were grown under conditions: 24°C temperature with a photoperiod of 12/12 h (day/night).

### Identification of *HcMYB* Genes

To identify HcMYB proteins in *H. coronarium*, 125 *Arabidopsis* (TAIR)^1^, and 127 tomato MYB protein sequences (SOL genome)^2^ were used as a query sequence in the *H. coronarium* genomic data (unpublished) and transcriptome data (Yue et al., 2015). A total of 286 candidate MYB proteins were obtained. Thereafter, SMART^3^ (Letunic et al., 2015) and NCBI Conserved Domain Database (Marchler-Bauer et al., 2015) with default parameters were used to confirm the presence of MYB domains. The reductant and false predicted sequences were excluded. A total of 253 *HcMYB* genes were finally identified in the *H. coronarium* genome and subsequently comprehensively analyzed. Through the ProtParam tool^4^, the physical and chemical parameters of *HcMYB* genes were measured. The N-terminal sequences of *HcMYB* genes were analyzed using prediction software (WoLF PSORT)^5^.

### Multiple Sequence Alignment, Phylogeny, and Synteny Analysis

Using the Clustal Ω program (Sievers et al., 2011), multiple sequence alignment of the putative *H. coronarium MYB* genes was analyzed with the default parameters. To construct an unrooted neighborhood phylogenetic tree of MYB TFs, the aligned protein sequences of AtMYBs, SlMYBs, OsMYBs, and HcMYBs were submitted to MEGA X (Kumar et al., 2018) with bootstraps set at 1,000 values. The syntenic relationship between *H. coronarium*, banana, pineapple, and rice was assessed using the MCScanX program (Tang et al., 2008).

### Gene Structure, Exon/Intron, Motif Prediction, and Analysis of *Cis*-Regulatory Elements

The distribution of exon/intron in *HcMYB* genes was visualized by submitting the corresponding coding and genomic sequences to the Gene Structure Display Server (GSDS)^6^ (Hu et al., 2015). Multiple Expectation Maximization for Motif Elicitation (MEME)^7^ (Bailey et al., 2015) was used to predict the conserved motifs in HcMYB proteins sequences. The MEME program was set at the following parameters: the maximum number of motifs at 10, while the other parameters were kept at default and then visualized in the web logo server^8^.

For promoters analysis, the 2 kb upstream genomic DNA sequences of *HcMYB* genes were retrieved from the *H. coronarium* genome and submitted to the PlantCARE database^9^ (Lescot et al., 2002) and verified in the PLACE databases^10^ (Higo et al., 1999).

### Subcellular Localization Analysis

For subcellular localization of selected MYB genes, HcMYB7, HcMYB8, HcMYB145, and HcMYB248 coding sequences with *Spe*I and *Nco*I restriction sites were fused with a green fluorescent protein (GFP) into the vector 35S_pro_:GFP (Wang et al., 2002). The transformation of *Arabidopsis thaliana* with 35S_pro_:GFP vectors and protoplasts isolation was performed following Yoo et al. (2007). The protoplasts were visualized 16–18 h after transformation through a laser scanning confocal microscope (LSCM). The primers used for GFP vector construction are listed in Supplementary Table 2.

### Yeast-One-Hybrid Assay

Transcriptional activity was determined using the yeast-one-hybrid system (Clontech, Takara) as described in the manufacturer’s protocol. The promoters of *HcTPS1*, *HcTPS3*, *HcTPS10*, and *HcBSMT2* were cloned into the vector pAbAi act as baits (*HcTPS1*-pAbAi, *HcTPS3*-pAbAi, *HcTPS10*-pAbAi, and *HcBSMT2*-pAbAi). The full-length of *HcMYB7*, *HcMYB8*, *HcMYB75*, *HcMYB79*, *HcMYB145*, *HcMYB238*, and *HcMYB248* were fused into the pGAL4 activation vector (pGADT7-AD) to generate pGADT7-HcMYB7, pGADT7-HcMYB8, pGADT7-HcMYB75, pGADT7-HcMYB79, pGADT7-HcMYB145, pGADT7-HcMYB238, and pGADT7-HcMYB248 as preys. The transformation of yeast cells and confirmation of positive interactions were performed as described in the Y1H Gold yeast strain manufacturer’s protocol (Clontech Takara).

### Yeast-Two-Hybrid (Y2H) Assay

The full-length sequences of HcMYBs (*HcMYB7*, *HcMYB8*, *HcMYB75*, *HcMYB79*, *HcMYB145*, *HcMY238*, and *HcMYB248*) were ligated into the vector pGADT7 (AD), and the coding sequences of HcJAZ1 was cloned into the vector pGBKT7 (BD). For the Y2H assay, the AH109 cells containing both BD and AD were placed on the SD/-Leu/-Trp medium for 3 days at 30°C. The empty vector pGADT7 was used as blank control. The transactivation activity was affirmed via yeast growth on the aforementioned plates. Yeast colonies expressing MEL1 turn blue with the addition of X-α-Gal substrate (Clontech, TaKaRa) because MEL-1 encodes α-galactosidase. Primers used are listed in Supplementary Table 2.

### Virus-Induced Gene Silencing (VIGS)

VIGS was conducted in the barley stripe mosaic virus (BSMV) system (Renner et al., 2009; Yuan et al., 2011). A 250- to 300-bp amplicon of *HcMYB7*, *HcMYB8*, *HcMYB145*, *HcMYB238*, and *HcMYB248* genes were inserted in pCaBSγ vector at *Apa*I restriction site making pCaBSγ:*HcMYB7*, pCaBSγ:*HcMYB8*, pCaBSγ:*HcMYB145*, pCaBSγ:*HcMYB238*, and pCaBSγ:*HcMYB248* constructs for corresponding genes supersession. The constructs were then transformed into *Agrobacterium tumefaciens* strain EHA105. For infiltration assays, the *A. tumefaciens* culture was suspended in infiltration buffer (10 mM MES; pH 5.6, 10 mM MgCl_2_, and 0.1 mM acetosyringone) at OD_600_ of 1. The solution was applied to the flowers at the D1 stage (bud stage). Vacuum infiltration was performed by immersing the flowers in the bacterial suspension. After the release of the vacuum, the flowers were washed in deionized water, placed into an MS medium liquid culture, and then maintained with a 12/12 h light/dark cycle at 16°C for 4–5 days. The total floral volatile compounds were collected and analyzed at the full-bloom stage by GC-MS. The experiment was performed in three to five biological replicates.

### GC-MS Analysis of Floral Volatiles

The whole flower was placed in a glass bottle (500 ml) with the addition of an internal standard. After 30 min, a PDMS fiber was injected for 30 min to trap volatile followed by insertion into a GC-MS system (Agilent) for volatile analysis as described previously (Ke et al., 2019). The floral volatiles were measured at the full-bloom stage via GC-MS as described previously (Yue et al., 2014; Abbas et al., 2019, 2020).

### RNA Isolation, cDNA Synthesis, and RT-qPCR

Total RNA extracted and cDNA was synthesized as described previously (Abbas et al., 2020). For RT-qPCR, 20 μl reaction system comprising 10 μl iTaq^TM^ Universal SYBR Green Supermix (BIO-RAD), 2 μl of cDNA, 0.4 μl of forward and reverse primers each, and 7.2 μl of ddH_2_O was performed in an ABI 7500 Fast Real-Time PCR System (Applied Biosystems, United States). GAPDH was used for normalization of data and the 2^–ΔΔ^*^C^*_T_ method was used for relative expression analysis (Livak and Schmittgen, 2001). All the reactions were performed in triplicate. The primers used for RT-qPCR are listed in Supplementary Table 2.

### Statistical Analysis

SPSS 19.0 program (SPSS Inc., Chicago, IL, United States) was used for statistical analysis and Student’s *t*-test. Data are presented as the mean ± SD and *p* < 0.05 (*n* = 3).

## Results

### Identification of the *HcMYB* Genes in *Hedychium coronarium*

A total of 253 potential candidate *HcMYB* genes were identified in *H. coronarium* genome data. All genes were designated as *HcMYB1-HcMYB253* based on the chromosomal location. Among them, 27 HcMYB genes belong to 1R, six 3R, one 4R, and the rest are all R2R3 type. The HcMYB protein size and molecular mass range from 100 aa/11.37 kDa (HcMYB100) to 1,749 aa/192.53 kDa (HcMYB10). Similarly, the *pI* also varies greatly from 4.51 (HcMYB170) to 12.2 (HcMYB252), indicating their functional diversity in microenvironments. A similar pattern was observed in *Prunus persica* and *Phyllostachys edulis* (Zhang C. et al., 2018; Yang et al., 2019b). Moreover, *in silico* analysis revealed that HcMYB proteins were predicted to localize in the nucleus. The detailed information of *HcMYB* genes is provided in Supplementary Table 1.

### Phylogenetic Divergence of *HcMYB* Genes

**FIGURE 1:**
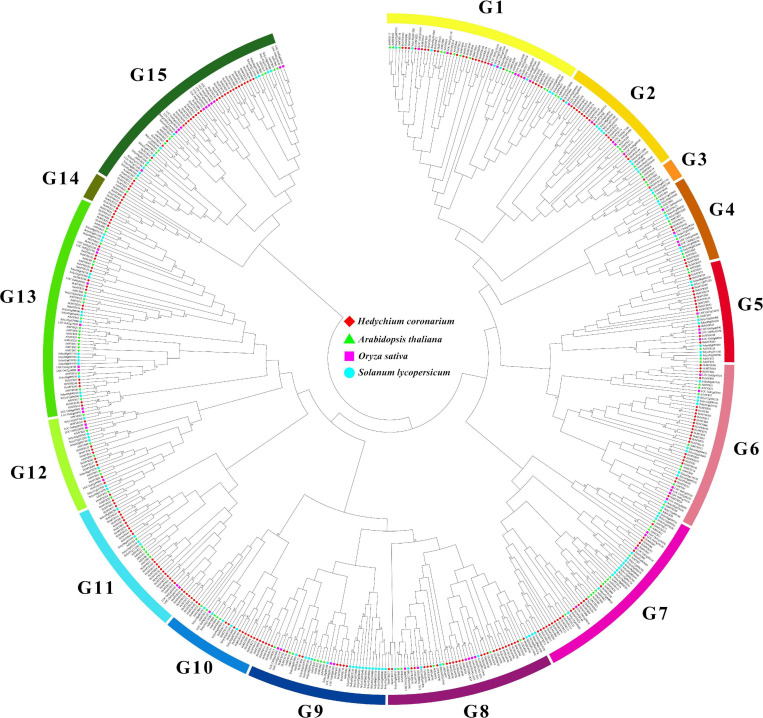
Phylogenetic analysis of MYB proteins among *H. coronarium*, *Arabidopsis*, tomato, and rice. Full-length amino acid sequences were aligned using ClustalX 2.1 program and unrooted NJ phylogenetic tree was generated using MEGA X with 1,000 bootstrap values. The bootstrap values of less than 50 were omitted. All MYB proteins are clustered into 15 subgroups (G1–G15) indicated by different colors.

The domain structure analysis showed that all *HcMYB* genes contain highly conserved typical SANT DNA-binding domains in their sequences, which is essential for their different regulatory interaction mechanism. The multiple sequence alignment of mostly *HcMYB* genes revealed that they possess 2R and 3R repeat signatures at the N-terminus, and seven candidate *HcMYB* genes were selected for multiple sequence alignment (Supplementary Figure 1). The seven candidate HcMYB proteins were selected for further characterization based on their expression pattern with flower scent emission. To assess the phylogenetic relationship among *MYB* genes in *H. coronarium* (253 *HcMYBs*), rice (66 *OsMYBs*), *Arabidopsis* (125 *AtMYBs*), and tomato (127 *SlMYBs*), an unrooted NJ phylogenetic tree was generated. All MYB proteins were divided into 15 distinct clades designed as G1–G15. Among them, subgroup G13 constitutes the largest group containing 64 MYB members of four different species followed by G15 (63), G1 (56), G7 (54), G8 (48), G6 (48), G7 (40), G11 (39), G5 (30), G12 (28), G2 (26), G10 (26), G4 (25), G14 (8), and G3 (6), respectively. Similarly, the maximum number of *HcMYB* genes were clustered into subgroup G15 (29) followed by G11 (27), G8 (27), G1 (25), G7 (23), G8 (23), G13 (20), G2, (15), G10 (15), G5 (13), G9 (12), G12 (10), G14 (7), G4 (5), and G3 (2), respectively. Interestingly, subgroup G14 contains eight MYBs from which seven belong to *H. coronarium* (Figure 1). Overall, all HcMYBs with AtMYB, OsMYB, and SlMYBs were unevenly clustered into all groups indicating their evolutionary divergence. The group of MYB genes in the same subclade may have a similar function. The phylogenetic tree analysis of only MYB proteins from *H. coronarium* revealed that 253 HcMYB proteins were distinctly grouped into 12 different clades (Supplementary Figure 2). Moreover, three groups (G3, G13, and G15) from four genome phylogeny, were missing. Moreover, the phylogenetic tree of 253 HcMYBs was also constructing with the previously identified scent-related MYB TFs (Supplementary Figure 3). The data showed that all HcMYBs along with previously identified scent-related MYBs were clustered into five distinct clades (G I-G V). The maximum number of scent-related MYB proteins were clustered in G II (11) followed by G V (5), G III (2), and G I (1), respectively. Furthermore, the selected seven-candidate HcMYB proteins were also grouped in different clades. HcMYB79 was clustered in G I, which included AtMYBL2, and HcMYB75/145/238 was found in G II, which contains the majority of scent-related MYBs (AtMYB2/21/24, FaEOBII, PhEOBI, PhEOBII, FvEOBII, PsMYB26, AmMYB305/340, and NiMYB3005). Similarly, HcMYB248 was found in G III, which includes AtMYB42 and PhODOI, while HcMYB7/8 was clustered in G V which includes AtMYB4/7, FaMYB1/10, PhMYB4, and PtMYB14 (Supplementary Figure 3).

### Gene Structure and Motif Analysis of *HcMYB* Genes

To investigate the functional diversification of HcMYB proteins, 10 conserved motifs (motifs 1–10) were identified in the MEME server (Supplementary Figure 4). The majority of the HcMYB proteins within the subclade had a similar composition of motifs, while variation was observed among different subclades. The motifs 1, 2, and 3 were the most conserved and appear in most of the HcMYB proteins. All the HcMYB proteins contain two or more than two motifs except HcMYB185 which only has motif 2 (Supplementary Figure 5). HcMYB23, HcMYB40, HcMYB47, HcMYB55, HcMYB110, HcMYB126, HcMYB168, and HcMYB253 proteins contained motif 3 and motif 8; HcMYB31, HcMYB67, HcMYB91, HcMYB121, and HcMYB179 proteins contained motif 1 and motif 2. HcMYB proteins containing motif 9 and motif 10 belong to subgroup G10 and G9, respectively. Interestingly, some HcMYB proteins contain the repetition of motif 3 and motif 8 and fall into subgroup G2 indicating their involvement in a specific function (Supplementary Figures 2, 5B).

Gene structure analysis was performed to better understand the expansion of *HcMYB* genes in *H. coronarium*. The number of exon/intron ranges from 0 to 21. Gene structure analysis revealed that five *HcMYB* (*HcMYB27*, *HcMYB87*, *HcMYB185*, *HcMYB217*, and *HcMYB249*) genes were intron-less, while *HcMYB10* have a maximum of 21 introns (Supplementary Figure 5C). Moreover, *HcMYB89* has the longest intron, and the majority of them (82%) were disrupted by two or three introns. In General, the *HcMYB* genes with the same number of introns were grouped into a similar subclade.

### Chromosomal Distribution and Duplication Events Among *HcMYB* Genes

**FIGURE 2:**
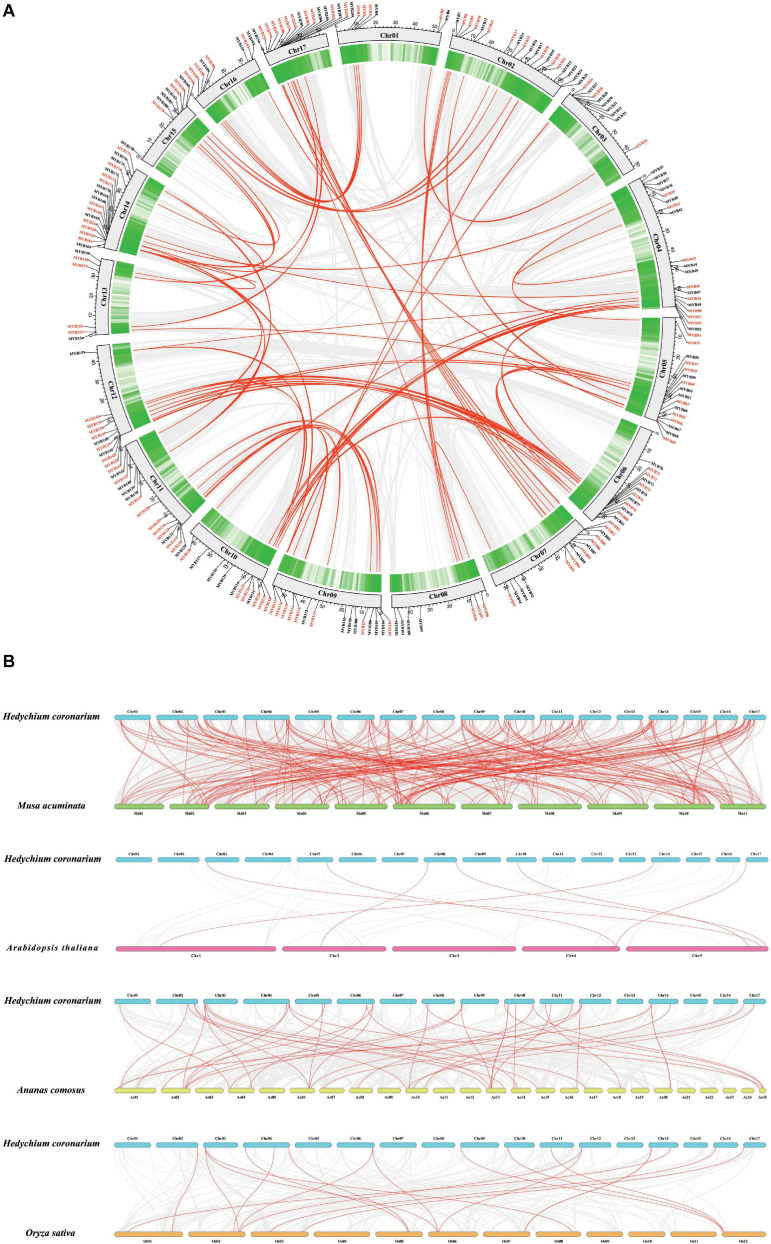
Synteny and collinearity analysis of MYB gene family. **(A)** Circos plot of MYB genes in *H. coronarium* genome. Segmental duplication of MYB gene pairs is indicated by red color in *H. coronarium*. **(B)** Colinearity plot of *HcMYB* genes between *Musa acuminata* and three other representative plant species. Gray lines in the background showed collinear blocks within *H. coronarium* and other plant genomes, while red lines indicate syntenic MYB gene pairs.

The chromosomal location of *HcMYBs* was performed to investigate the genomic distribution of the MYB gene family in *H. coronarium*. The results revealed an uneven distribution of *HcMYB* genes in the chromosomes (Figure 2A). In total, 6 *HcMYB* genes were found on chromosomes 1 and 13, 7 on chromosomes 8 and 16, 9 on chromosomes 3 and 15, 11 on chromosomes 10 and 12, 12 on chromosome 6, 14 on chromosomes 7 and 11, 15 on chromosomes 5 and 9, 16 on chromosome 17, 18 on chromosome 14, and 19 on chromosome 2. The maximum number of *HcMYB* genes (20) were presented on chromosome 4. The majority of *HcMYB* genes were observed on the top and bottom of the chromosomes, while rarely found in the middle of the chromosome.

Gene duplications play a crucial role in the evolution and expansion of gene families in plants. It was found that *H. coronarium* genome contained 62 HcMYB segmental duplications (Figure 2A). The maximum number of segmental duplication events was observed on chromosomes 14 and 17, while a low number of duplication events were found on chromosome 3. Moreover, three tandem duplication pairs were also identified on chromosome 17 in *H. coronarium* genome. The *Ka/Ks* (synonymous/non-synonymous) values of all segmentally and tandemly duplicated *HcMYB* gene pairs had less than 1, indicating that they evolved under the pressure of purifying selection (Figure 2A and Supplementary Table 3). The average *Ka/Ks* value of tandem duplication genes (0.57) was higher than that segmented duplication genes (0.27). Moreover, the segmental and tandem duplication gene events occurred about 47 Mya (million years ago), implying that duplication events play a crucial role in functional and evolutional divergence.

To determine the evolutionary mechanism of *HcMYB* genes, comparative synteny maps of *H. coronarium* associated with rice, pineapple, Arabidopsis, and banana were constructed (Figure 2B). A total of 7 ortholog MYB gene pairs between *H. coronarium* and *Arabidopsis*, 19 orthologs between *H. coronarium* and rice, 44 orthologs between *H. coronarium* and pineapple, and 193 orthologs between *H. coronarium* and banana were identified (Figure 2B and Supplementary Tables 4–7). The number of HcMYB-MaMYB was greater than HcMYB-AtMYB, HcMYB-OsMYB, and HcMYB-AcMYB, indicating the closer evolutionary relationship between *H. coronarium* and *Musa accuminata* and their divergence from the common monocot ancestor. Interestingly, HcMYB29 and HcMYB194 collinear gene pairs were present among four species. HcMYB29 was homologous pair of AT5G65230.1 (*A. thaliana*), LOC_Os02g51799.1 (*O. sativa*), Aco002802.1 (*A. comosus*), and GSMUA_Achr10T24560 (*M. acuminata*). Similarly, HcMYB194 was collinear gene pair of AT4G37780.1, LOC_Os03g56090.1, Aco004978.1, and GSMUA_Achr5T01930. In short, the aforementioned two collinear gene pairs were found among four species genomes, implying their evolutionary mechanism and specific function in *H. coronarium*.

### *Cis-*Regulatory Elements and *HcMYB* Targeting miRNAs

A 2-kb upstream promoter region of *HcMYB* genes was scanned for *cis*-regulatory elements analysis. The data showed that MeJA responsiveness elements are the most common ones, which were found in 210 *HcMYB* gene promoters. Other *cis*-acting regulatory elements found in the promoter region of HcMYB genes were ABARE (208), ERE (180), GARE (144), MBS (128), Auxin responsive (120), SARE (99), and low-temperature responsiveness elements (96) (Supplementary Figure 6). The detailed information regarding the *cis*-elements present in the promoter region of *HcMYB* genes is given in Supplementary Table 8. Previous studies showed that miRNA play key roles in plant secondary metabolism. In *A. thaliana*, miR858 encodes regulatory peptides and controls flavonoid biosynthesis and development (Sharma et al., 2020). Similarly, miR156 was involved in the regulation of anthocyanin biosynthesis in poplar (Wang et al., 2020). The miRNA prediction analysis showed that *HcMYB* genes were targeted by different families of miRNA. About 37 *HcMYB* genes were mainly targeted by miR156, miR157, miR158, miR160, miR167, miR168, miR169, miR172, miR319, miR854, and miR858. The *HcMYB* genes were commonly targeted by miR156, miR167, and miR319 (Supplementary Table 9). Interestingly, *HcMYB187*, *HcMYB166*, and *HcMYB157* were targeted by both miR167 and miR319. The majority of *HcMYB* genes (42) were targeted by miR858.

### Expression of *HcMYB* Genes at Different Organs and Flower Developmental Stages

**FIGURE 3:**
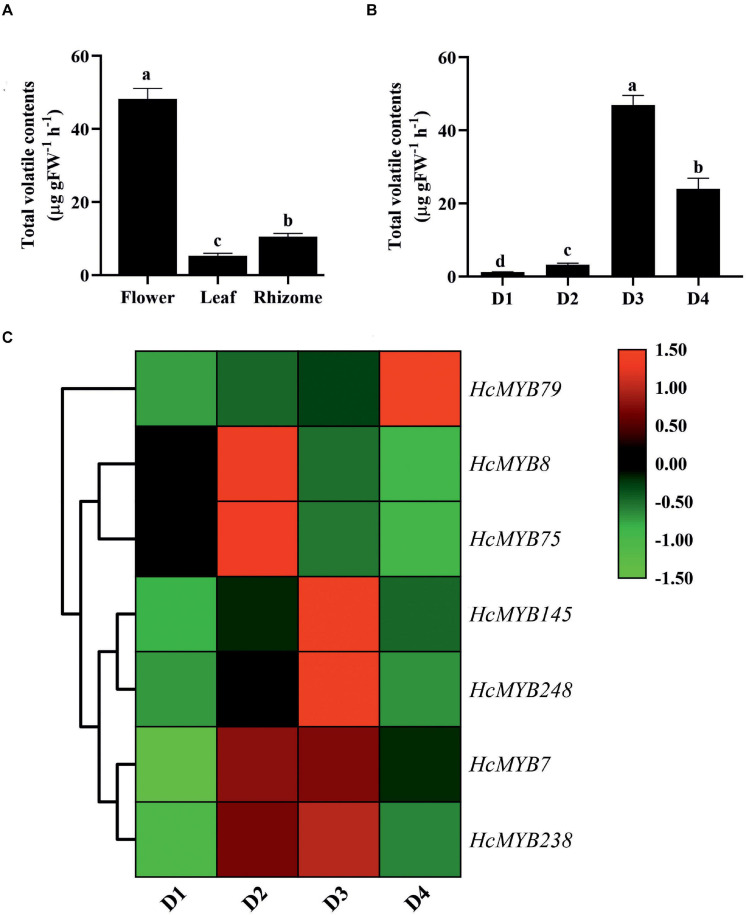
The total volatile contents and expression levels of candidates *HcMYB* genes. **(A)** The total volatile contents emitted from flower, leaf, and rhizome. **(B)** The total floral volatiles released during different flower developmental stages (D1: squaring stage; D2: half open stage; D3: full-bloom; D4: senescence stage). **(C)** Differential expression patterns of selected candidate *HcMYB* genes during four different flower developmental stages. The heat map was generated with the relative expression RT-qPCR data of candidate *HcMYB* genes. The red (upregulated expression levels) and green (downregulated expression levels) color represent the log^2^ transformed expression values.

The differential expression patterns of *HcMYB* genes in different organs and flower developmental stages were analyzed using the RNA sequencing data (Yue et al., 2015; Supplementary Figure 7). The expression pattern of *HcMYB* genes was grouped into three clusters. The cluster I showed a group of *HcMYB* genes, which had preferential expression pattern in flowers. Similarly, cluster II and cluster III represent the member of *HcMYBs*, which showed their high expression levels in rhizome and leaves, respectively (Supplementary Figure 7A). Interestingly, *HcMYB7*, *HcMYB8*, *HcMYB75*, *HcMYB145*, *HcMYB238*, and *HcMYB248* from cluster I had the highest expression level in the flower. Some members of the cluster I also showed their spatial expression both in flower and rhizome including *HcMYB79*, *HcMYB128*, *HcMYB162*, *HcMYB130*, *HcMYB108*, *HcMYB199*, and *HcMYB208.* The members of *HcMYB* genes in cluster II showed high expression in the rhizome, while a few genes from cluster III showed high expression both in flowers and leaves and some in leaves and rhizomes. However, the majority of genes from cluster III showed their high expression in leaves. The amount of volatile contents released from flowers was maximum, while less quantity was observed in leaves and rhizomes (Figure 3A). Previous studies showed that tissue-specific expression is important for gene functioning (Sonawane et al., 2017). The data indicate the group of *HcMYBs* present in cluster I might be involved in floral scent formation, while cluster II and cluster III in the functioning of vegetative organs (rhizome and leaves).

In *H. coronarium*, the process of floral scent formation is associated with flower development. The emission quantity of floral volatiles was low at the bud stage and substantially increase with the flower development peak at the blooming period and declined at the senescence stage (Figure 3B). To clarify the functioning of *HcMYBs* during flower development stages, their expression level at three stages was divided into three clades via a heat map (Supplementary Figure 7B). Several *HcMYB* genes showed high expression levels at the D1 stage followed by D4 and D6, respectively. However, some *HcMYBs* (*HcMYB34*, *HcMYB51*, *HcMYB181*, and *HcMYB232*), which belong to cluster I, showed a substantial increase in expression level with the developmental stages. The members of *HcMYB* genes from cluster II showed diverse expression levels. The majority of genes showed a high expression level at the D4 stage only, although *HcMYB5*, *HcMYB62*, *HcMYB106*, *HcMYB111*, *HcMYB240*, and *HcMYB245* from cluster II showed high expression both at D1 and D4 stage. Likewise, within-cluster II, some *HcMYB*s (*HcMYB16*, *HcMYB94*, *HcMYB203*, *HcMYB208*, *HcMYB218*, and *HcMYB221*) showed specific high expression both at D4 and D6 stage only (Supplementary Figure 7B). Interestingly, *HcMYB*22, *HcMYB*55, and *HcMYB*112, within-cluster III, showed a high expression at the D1 stage, with no expression at the D4 stage and dramatically peak at the D6 stage.

Based on transcriptome data, seven *HcMYB* genes were selected and their spatial expression pattern was validated via RT-qPCR at four different flower development stages (Figure 3C). The expression level of *HcMYB79* increased with the flower development and peak at the senescence stage, while *HcMYB8* and *HcMYB75* showed a high expression level at the D2 stage (half-open). However, *HcMYB7*, *HcMYB145*, *HcMYB238*, and *HcMYB248* showed a dramatic increase in the expression level from D2 to D3 stage. Moreover, the expression of candidate *HcMYB* genes correlates with the emission of total volatile contents (Supplementary Figure 8). The expression level of *HcMYB* genes significantly altered with the flower developmental process, suggesting their potential role during the development of flower and floral scent emission.

### Expression of *HcMYBs* in Response to Hormone Stresses

**FIGURE 4:**
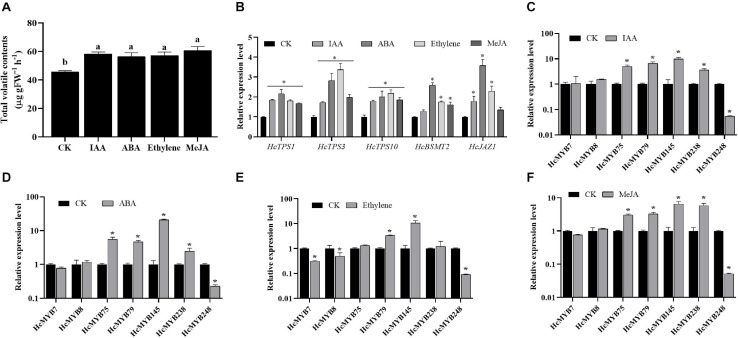
Relative expression level of candidate *HcMYB* genes and emission of total floral volatiles under phytohormone stresses. **(A)** Total floral volatiles contents emitted during phytohormone treatments. **(B)** mRNA levels of key structural volatile biosynthesis genes upon phytohormone stresses. **(C)** IAA: (Inode-3-acetic acid), **(D)** ABA: (abscisic acid), **(E)** ethylene, **(F)** MeJA: jasmonic acid methyl ester. Error bars indicate SD of three biological replicates and asterisk represents significant differences (^∗^*p* < 0.05).

Plant hormones play several functions in growth and development and are involved in different signaling pathways. Auxin, abscisic acid, ethylene, and jasmonates are the key hormones playing an essential role in flower development and senescence. The amount of floral volatile contents was increased by 27.6, 23.9, 25.5, and 33.1% under IAA, ABA, ethylene, and MeJA treatment, respectively (Figure 4A). The expression levels of selected *HcMYBs* were analyzed via RT-qPCR under the abovementioned treatments. The data showed that mRNA levels of *HcMYB75*, *HcMYB79*, *HcMYB145*, and *HcMYB238* were upregulated under auxin treatments, while *HcMYB248* was downregulated (Figure 4C). Under ABA treatment, *HcMYB145* expression level was increased by 19-fold followed by *HcMYB75*, *HcMYB79*, and *HcMYB238*, respectively. However, the expression level of *HcMYB248* was reduced (Figure 4D). For ethylene treatment, *HcMYB79* and *HcMYB145* were significantly upregulated; however, *HcMYB7*, *HcMYB8*, and *HcMYB248* were downregulated (Figure 4E). The expression levels of *HcMYB75*, *HcMYB79*, *HcMYB145*, and *HcMYB238* were upregulated when treated with MeJA, while *HcMYB248* was downregulated (Figure 4F). Moreover, mRNA levels of key structural biosynthesis genes (*HcTPS1*, *HcTPS3*, *HcTPS10*, and *HcBSMT2*) were also upregulated upon phytohormones stresses (Figure 4B). These results indicate that *HcMYB* genes respond to various stresses and play crucial roles through crosstalk with different hormones.

### Subcellular Localization of HcMYB Proteins

**FIGURE 5:**
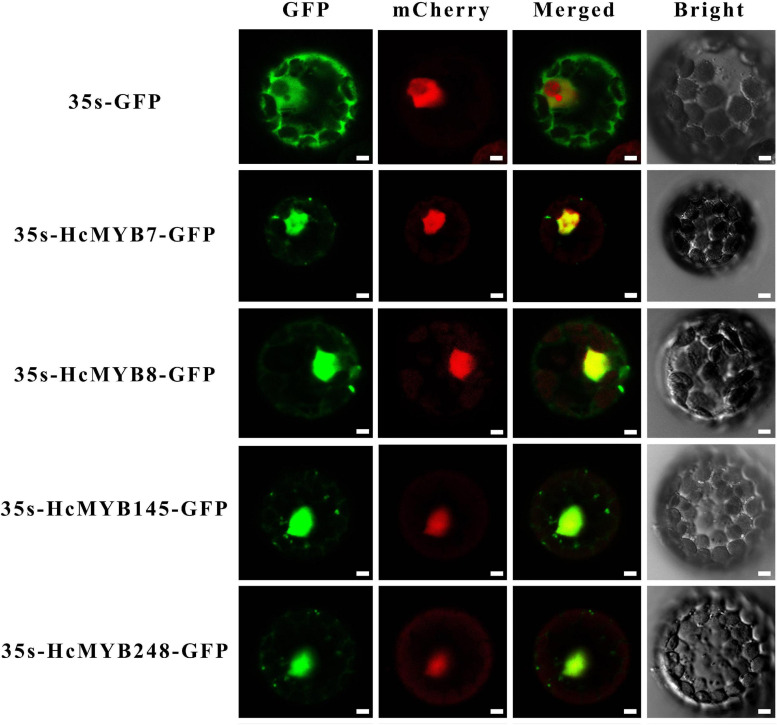
Nuclear localization of HcMYB7/8/145/248 proteins in Arabidopsis protoplasts. GFP; green fluorescent protein, mCheyy; nuclear marker, merged; combined mCheryy and GFP and bright field. Bars, 10 μm.

*In silico* subcellular localization of *HcMYB* genes revealed that all *H. corornarium* MYB genes are predicted to localize in the nucleus except HcMYB126, which is predicted to localize in the chloroplast (Supplementary Table 1). To experimentally validate the predicted localization, four *HcMYB* genes (*HcMYB7*, *HcMYB8*, *HcMYB145*, and *HcMYB248*) were selected for analysis in *Arabidopsis* protoplast. The NLS-mCherry was applied in each transformed design to act as a marker for nuclear localization. The results revealed that HcMYB7, HcMYB8, HcMYB145, and HcMYB248 proteins were localized to the nucleus (Figure 5).

### Suppression of *HcMYB* Genes Altered the Emission of Main Floral Volatile Compounds

**FIGURE 6:**
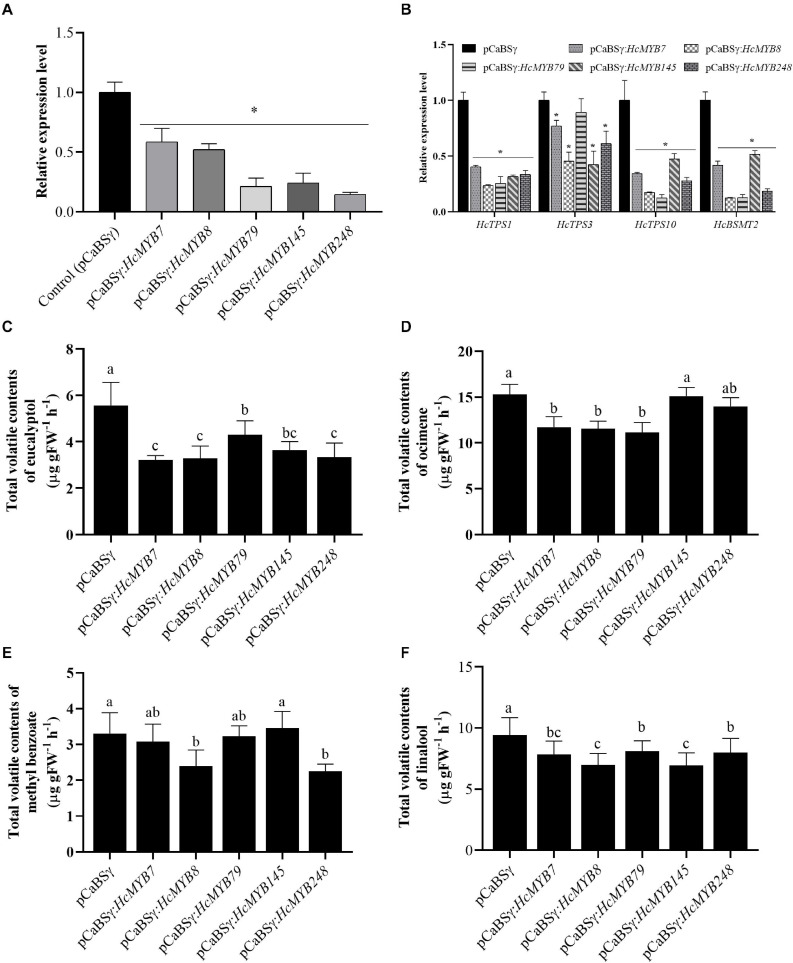
HcMYB7/8/79/145/248 silencing changes the amount of main floral volatile contents and expression of key structure genes in *H. coronarium* flowers. **(A)** RT-qPCR analysis of *HcMYB7/8/79/145/248* mRNA levels in BSMV silenced and control flowers. **(B)** mRNA levels of bottom structural volatile synthesis genes in BSMV silenced flowers compared with control. **(C)** GC-MS analysis of volatile contents of eucalyptol, **(D)** ocimene, **(E)** methyl benzoate, and **(F)** linalool after silencing of *HcMYB7/8/79/145/248* genes. Data are presented as the mean ± SEM (*n* = 3). Asterisk indicates significant differences [Student’s *t*-test, ^∗^(*P* ≤ 0.05)] between treatments and control.

To understand the potential involvement of *HcMYB* genes in the floral scent formation, a virus-induced gene silencing system was performed for *HcMYB7*, *HcMYB8*, *HcMYB79*, *HcMYB145*, and *HcMYB248*. The data showed that the mRNA expression level of *HcMYB7*, *HcMYB8*, *HcMYB79*, *HcMYB145*, and *HcMYB248* was decreased 41.4, 47.8, 78.7, 75.8, and 85.3%, respectively (Figure 6A), relative to the control flowers. The silencing of *HcMYB* genes in flowers also downregulated the expression level of key structural volatile biosynthesis genes. In the *HcMYB7*-silenced flower, the expression level of *HcTPS1*, *HcTPS3*, *HcTPS10*, and *HcBSMT2* was downregulated up to 59.7, 23.1, 65.7, and 58.3%, respectively, compared to control. The silencing of *HcMYB8* in *H. coronarium* flowers, downregulated the expression level of *HcTPS1*, *HcTPS3*, *HcTPS10*, and *HcBSMT2* by 76.5, 54.5, 82.8, and 87.7%, respectively. Alike, silencing of *HcMYB79* significantly downregulated the expression levels of *HcTPS1* (74.4%), *HcTPS10* (87.6%), and *HcBSMT2* (87.4%). The expression level of *HcTPS1* (68.3%), *HcTPS3* (57.7%), *HcTPS10* (52.6%), and *HcBSMT2* (48.3%) was significantly suppressed in the *HcMYB145*-silenced flowers. Furthermore, the mRNA levels of *HcTPS1* (66.5%), *HcTPS3* (38.8%), *HcTPS10* (72.1%), and *HcBSMT2* (81.4%) were significantly changed in the *HcMYB248*-silenced flowers (Figure 6B).

The effect of silencing of *HcMYB* genes on the floral volatile profile of *H. coronarium* was assessed. It was observed that eucalyptol contents were decreased 42.2, 40.8, 22.6, 34.6, and 39.8% in the *HcMYB7*, *HcMYB8*, *HcMYB79*, *HcMYB145*, and *HcMYB248*-silenced flowers, respectively (Figure 6C). Similarly, the silencing of *HcMYB7*, *HcMYB8*, and *HcMYB79* significantly decreased the accumulation of ocimene by 23.3, 24.4, and 27.1%, respectively. However, no significant change in volatile contents was found in the *HcMYB145* and *HcMYB248*-silenced flowers (Figure 6D). The volatile contents of methyl benzoate were significantly decreased by 27.6 and 31.9% in the *HcMYB8* and *HcMYB248*-silenced flowers, respectively, while silencing of *HcMYB145* did not affect the level of methyl benzoate (Figure 6E). Furthermore, the contents of linalool were decreased by 16.9, 26, 13.9, 26.2, and 15.1% in the *HcMYB7*, *HcMYB8*, *HcMYB79*, *HcMYB145*, and *HcMYB248*-silenced flowers, respectively (Figure 6F). The data showed that these *HcMYB* genes play a crucial role in the floral scent formation in *H. coronarium.*

### HcMYBs Binds to the Promoters of Key Structural Volatile Biosynthesis Genes

**FIGURE 7:**
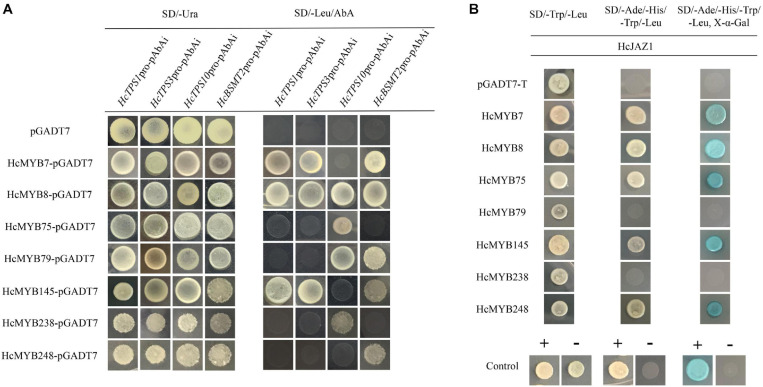
Candidate HcMYB proteins binds to the promoters of bottom structural genes. **(A)** The interaction was determined on SD medium lacking leucine (Leu) in the presence of aureobasidin A (-Leu + AbA). AD was used as a control. **(B)** HcMYB7/8/75/79/145/238/248 interacts with HcJAZ1 protein by yeast two-hybrid assay. The vectors were transformed into yeast strain Y2HGold followed by the screening of transformants via SD/-Trp/-His/-Ade + X-α-gal media. Yeast cells transformed with pGBKT7–53 + pGADT7-T, pGBKT7-Lamin + pGADT7-T, or pGADT7-T- pGBKT7-HcJAZ1 were included as negative or positive controls.

Promoter sequences analysis of *HcTPS1* (1,131), *HcTPS3* (1,557), *HcTPS10* (867), and *HcBSMT2* (1,131) revealed that they encompass 6, 4, 3, and 13 copies of MYB-core binding motifs, respectively (Supplementary Table 10). To examine that the identified HcMYB regulators directly bind or not to the promoters of key volatile floral scent biosynthesis genes (*HcTPS1*, *HcTPS3*, *HcTPS10*, and *HcBSMT2*) in *H. coronarium*, a Y1H assay was conducted. It was observed that the bait strains harboring *proHcTPS1* and *proHcTPS3* grow well on gained aureobasidin A antibiotic SD-Leu medium co-expression with HcMYB7, HcMYB8, and HcMYB145. Moreover, bait strains harboring *proHcTPS10* gained aureobasidin A antibiotic resistance upon expression of HcMYB8, HcMYB75, HcMYB79, and HcMYB238. Furthermore, bait strains harboring *proHcBSMT2* gained aureobasidin A antibiotic resistance upon expression of HcMYB7, HcMYB8, HcMYB79, HcMYB145, and HcMYB248 (Figure 7A).

### HcMYBs Interacts With JAZ1 Protein

To further unravel the molecular mechanism of the involvement of HcMYBs in the regulation of floral aroma biosynthesis, a yeast-two-hybrid (Y2H) assay was performed. It was observed that pGADT7-T + pGBKT7-53 (positive control) along with yeast cells, which co-transformed with pGADT7-HcMYB7 + pGBKT7-HcJAZ1, pGADT7-HcMYB8 + pGBKT7-HcJAZ1, pGADT7-HcMYB75 + pGBKT7-HcJAZ1, pGADT7-HcMYB145 + pGBKT7-HcJAZ1, and pGADT7-HcMYB7248 + pGBKT7-HcJAZ1, grew well on SD plates lacking Trp, His, Ade, and Leu but in the existence of 5 mM 3-AT. On the other hand, yeast cells harboring pGBKT7-Lam + pGADT7-T (negative control), pGADT7-HcMYB79 + pGBKT7-HcJAZ1, and pGADT7-HcMYB238 + pGBKT7-HcJAZ1 did not grow (Figure 7B). These results indicated that HcJAZ1 may interact with five HcMYBs (HcMYB7, HcMYB8, HcMYB75, HcMYB145, and HcMYB248) in yeast.

## Discussion

*H. coronarium* is an important industrial, medicinal, and ornamental plant. The snowy white flowers emit a strong scent mainly composed of terpenes, benzoates, and some phenylpropanoids (Matsumoto et al., 1993; Fan et al., 2003, 2007; Báez et al., 2011). The MYB gene family is one of the biggest TF families playing key physiological and biochemical roles in the plant. The role of this family in *H. coronarium* is unknown. Taking the advantage of this and the availability of *H. coronarium* in our group, we performed a genome-wide analysis of the MYB gene family and its potential role in floral aroma production.

### Evolutionary Analysis of *HcMYB* Gene Family

The number of MYB genes varies among different species including 204 MYB TFs in *Arabidopsis thaliana*, 218, 244, 256, 127, 116, 122, and 85 MYBs from *Oryza sativa*, *Glycine max*, *Prunus persica*, *Solanum lycopersicum*, *Physcomitrella patens*, *Brachypodium distachyon*, and *Phyllostachys edulis*, respectively (Du et al., 2012; Katiyar et al., 2012; Li Z. et al., 2016; Chen S. et al., 2017; Zhang C. et al., 2018; Yang et al., 2019b; Pu et al., 2020). However, a total of 253 HcMYB genes were identified in *H. coronarium.* The *HcMYB* genes can be divided into 4 families, including 27 1R-MYB proteins, 219 R2R3-MYB proteins, 6 3R-proteins, and 1 4R-MYB protein. The presence of one 4R-MYB protein identified in *H. coronarium* was in line with previous findings in *Arabidopsis*, peach, pear, and Chinese jujube (Dubos et al., 2010; Lan et al., 2013; Li X. et al., 2016; Zhang C. et al., 2018). However, in some plants, more than one 4R-MYB protein have also been reported (Saha et al., 2016; Salih et al., 2016). In the current study, six 3R-MYB proteins were identified, which is inconsistent with other findings: 11 3R-MYB proteins in Chinese cabbage (Saha et al., 2016), 15 in cotton (Salih et al., 2016), and four in tomato and peach (Li Z. et al., 2016; Zhang C. et al., 2018). The differences in numbers are probably due to the differences in the evolution of plants.

Gene duplication plays a crucial role in the expansion and evolution of genes and accelerates the expansion of gene families (Davidson et al., 2013; Chen S. et al., 2017). The data showed that segmental and tandem duplication events occur unevenly on all chromosomes (Figure 2A). Sixty two *HcMYB* genes were identified as segmentally duplicated, while three *HcMYB* genes were observed as tandemly duplicated genes. Similar trends of MYB gene duplication pairs were observed in *Arabidopsis thaliana* (Cannon et al., 2004), *Solanum tuberosum* L. (Sun et al., 2019), *Citrus sinensis* (Liu et al., 2014), and *Ananas comosus* (Liu et al., 2017). The results indicate that segmental duplication events play key roles in the expansion of *MYB* genes compared to tandemly duplicated genes. The *Ka/Ks* ratios of 65 HcMYB replications suggested that this gene family undergo purifying selection, a clear indication of highly conserved evolution.

To gain more insight into the evolutionary relationship of *HcMYB* genes, a phylogenetic tree was built including MYB proteins from *Arabidopsis thaliana*, *Oryza sativa*, and *Solanum lycopersicum*. As shown in Figure 1, all MYB genes were distinctly grouped into 15 different clades from G1–G15. *HcMYB7* and *HcMYB8* genes fell into subgroup G11, which contains *AtMYB32*, *AtMYB3*, *AtMYB4*, *AtMYB6*, *AtMYB7*, and *AtMYB8*, which are involved in anthocyanin and flower development (Jin et al., 2000; Vimolmangkang et al., 2013; Fornalé et al., 2014). *HcMYB75* and *HcMYB145* with *AtMYB21*, *AtMYB24*, and *AtMYB78* were grouped in subclade G6, which had flower-specific expression and involved in plant secondary metabolism (Shin et al., 2002; Spitzer-Rimon et al., 2010; Medina-Puche et al., 2015). Similarly, *HcMYB79* was clustered in subgroup G1 with *AtMYB1*, *AtMYB44*, *AtMYB70*, and *AtMYB77*. The functional characterization of the abovementioned TFs revealed that they were involved in different abiotic stress responses (Jung et al., 2008). *HcMYB238* and *HcMYB248* were grouped into subclades G5 and G15, respectively. The results showed that the tandemly or segmentally duplicated genes were grouped into the same clade (Supplementary Figure 2). The prediction results showed that all *HcMYB* genes include the conserved MYB DNA-binding domain; however, some *HcMYB* genes also possess additional domains, such as *HcMYB6* containing a CS domain, which is involved in recruiting heat-shock proteins to multi-protein assemblies (Lee et al., 2004). Similarly, *HcMYB10* includes additional VHS-ENTH-ANTH, GAT, and Med15 superfamily (Supplementary Figure 6B). Moreover, the exon/intron structure and motif analysis of *HcMYB* genes was also performed. The data showed that the majority of *HcMYB* genes contained motif 1, motif 2, and motif 3. Furthermore, *HcMYB* genes (∼82%) had two to three number of introns, which is consistent with previous findings (Du et al., 2012; Liu et al., 2020). In General, the number of motifs and intron in the same clade were similar, while variations were observed in a few clades, implying the functional redundancy of these genes. The colinearity relationship of *HcMYB* genes revealed high levels of collinearity with *Musa acuminata* MYB genes followed by *Ananas comosus*, *Oryza sativa*, and *Arabidopsis thaliana* implying that these MYB genes probably come from a common ancestor (Figure 2B). Two colinear genes (*HcMYB29* and *HcMYB194*) were observed, which exist in all the plant’s genomes, indicating that they are probably associated with the evolutionary processes.

### Expression of *HcMYB* Genes Correlate With the Flower Development and Scent Emission

MYB transcription factors play key roles in regulating plant secondary metabolites. The production, as well as emission of floral volatile compounds, is developmentally regulated and peak at the bloom stage (Muhlemann et al., 2014; Abbas et al., 2017). Likewise, in *H. coronairum*, the maximum floral volatile contents were emitted at the full-bloom flower (Figure 3A). As shown in Figure 5, the group of *HcMYB* genes in cluster I showed their preferential expression in flowers. Moreover, the RNA-seq data of different flower development stages revealed their higher expression pattern with the flower development stage, implying their potential involvement in the production and emission of floral aroma. In *Syringa oblata*, two R2R3-MYB transcription factors showed a high expression pattern during the flower development, which is similar in strawberry and petunia (Van Moerkercke et al., 2011; Medina-Puche et al., 2015). The expression pattern of selected *HcMYB* genes at four different flower development stages showed a substantial increase in the mRNA level consistent with the flower development stage (Figure 3). Interestingly, the key structural genes (*HcTPS1*, *HcTPS3*, *HcTPS10*, and *HcBSMT2*) also showed flower-specific expression consistent with the production of high volatile contents at the full-bloom stage (Yue et al., 2015). Moreover, the formation of floral volatile compounds was higher in the flower than in leaves and rhizome. Likewise, *Pinus taeda MYB14* (*PtMYB14*) regulates the isoprenoid-oriented response, which encourages the accumulation of sesquiterpene (Bedon et al., 2010). The *FaEOBII, FaEOBI*, and *ODO1* from strawberry also showed flower specific expression pattern and was involved in the regulation of eugenol (Verdonk et al., 2005; Spitzer-Rimon et al., 2010; Van Moerkercke et al., 2011, 2012; Medina-Puche et al., 2015). *PpMYB15* and *PpMYBF1* from *Prunus persica* had flower-specific expression and involved in the regulation of flavanol biosynthesis (Cao et al., 2018). The data indicate that the *HcMYB* genes potentially regulate the floral aroma production during flower development.

### *HcMYB* Genes Play Key Roles in Floral Scent Formation by Binding to the Promoters of Key Structural Genes

*HcTPS1*, *HcTPS3*, *HcTPS10*, and *HcBSMT2* are the bottom structural genes that are involved in the majority of the floral aroma production in *H*. *coronarium* (Yue et al., 2015) and contained MYB-binding elements in the promoter regions (Supplementary Table 10). The volatile organic compounds are diverse and play significant roles throughout the plant lifespan. The yeast-one-hybrid assay results showed that *HcMYB7/145* can transactivate the promoter of *HcTPS1*, *HcTPS3*, and *HcBSMT2*, while *HcMYB75/238* activates the promoter of *HcTPS10*. Moreover, *HcMYB8* transactivates the promoter of *HcTPS1*, *HcTPS3*, *HcTPS10*, and *HcBSMT2*, while *HcMYB248* activates the promoter of *HcBSMT2*. Furthermore, *HcMYB79* binds to the promoters of *HcTPS10* and *HcBSMT2* (Figure 7A). *ODO1*, *EOBI*, and *EOBII* from *Petunia* regulate the biosynthetic pathway of benzenoids by regulating the transcript level of several key structure genes [CHORISMATE MUTASE (*CM*), *PHENYLALANINE AMMONIA LYASE* (*PAL*), 3-deoxy-d-arabino-heptulosonate-7-phosphate synthase (*DAHPS*), and *S*-adenosyl Met Synthase (*SAMS*)]. *EOBI* not only directly binds but also activates the promoters of *PAL*, *ODO1*, and ISOEUGENOL SYNTHASE (*IGS*) to regulate aroma production (Spitzer-Rimon et al., 2010, 2012; Saha et al., 2016). In strawberry, *FaMYB10* regulates several structural genes and is involved in the biosynthesis of flavonoids and phenylpropanoids (Medina-Puche et al., 2014). Our findings indicate that HcMYB7/8/75/79/145/238/248 proteins directly activate these structural genes and are localized to the nucleus (Figures 5, 7B). Notably, the HcMYB8 protein was capable of activating the promoters of different volatile biosynthesis genes. *AtMYB21* and *AtMYB24* from *Arabidopsis* were involved in the production of sesquiterpenes (Tholl et al., 2005). In spearmint, *MsMYB* binds to the promoter region of geranyl diphosphate synthase (*GPPS*) and negatively regulates the production of monoterpenes by suppressing the activity of *GPPS* (Amarr et al., 2017). Moreover, to investigate the involvement of *HcMYB7/8/79/145/248* in floral scent formation, the activity of these genes was suppressed via gene silencing (Figure 6). There was a significant decrease in the floral volatile contents and suppressed the expression of scent-related key genes in the *HcMYB7/8/79/145/248*-silenced flowers (Figure 6B). Likewise, in petunia, suppression of *ODO1* downregulated the expression of numerous scent-related genes (Spitzer-Rimon et al., 2012). Overexpression of *Vitis vinifera VvMYB5b* in tomato encourages the accumulation of β-carotene (Mahjoub et al., 2009). *LcMYB5* directly activated the expression of key structural genes and was involved in anthocyanin biosynthesis in litchi (Lai et al., 2019). Less information is available regarding the transcriptional regulatory mechanism of MYB genes in the floral biosynthetic pathway in non-model fragrance plants, such as *H. coronarium*.

The transcriptional complexes formed by protein–protein interactions between MYBs and other proteins are necessary to control gene expression (Ali and Baek, 2020). In *Arabidopsis*, AtJAZ proteins interact with MYBs, modulating the expression of key enzyme genes, such as C4H, in the anthocyanin biosynthesis pathway (Qi et al., 2011). Similarly, AtJAZ protein interacts with MYB21/24 to affect jasmonate-regulated stamen development (Xie et al., 2016). Further studies also revealed that JAZ proteins interact with the WD-repeat/bHLH/MYB complexes to regulate anthocyanin accumulation (Payne et al., 2000). In *Fagopyrum tataricum*, four MYB transcription factors (FtMYB13/14/15/16) interact with FtJAZ1 to regulate the accumulation of rutin (Zhang K. et al., 2018). In *S. lycopersicum*, application of JA induces defense protein accumulation and volatile emissions in plants (Degenhardt et al., 2010). Several JA-responsive genes are involved in the biosynthesis of numerous clades of secondary metabolism and responses to wounding and herbivores (Wu and Baldwin, 2010). In lima bean, it has been reported that the production of volatile compounds is regulated by JA (Dicke et al., 1999). In our study, the Y2H assay revealed that HcMYB7/8/75/145/248 interact with HcJAZ1 protein (Figure 7B). The volatile organic compounds are diverse and play significant roles in the plant lifespan. Our results will provide new insight into the mechanism of floral aroma production and role of MYB TFs in JA signaling transduction in non-model fragrance plants.

### *HcMYB* Gene Response to Phytohormone Stresses

Phytohormones including auxin, ABA, ethylene, and MeJA are key hormones that regulate various plant developmental processes including flower development and senescence (Zubo et al., 2011; Wils and Kaufmann, 2017; Ke et al., 2019). Several *cis*-regulatory elements related to hormone response were predicted in the promoters of *HcMYB* genes, implying that *HcMYB* genes respond to hormones. The data showed that MeJA responsive elements were the most common in the promoter sequences of *HcMYB* genes followed by ABARE, ERE, GARE, auxin, SARE, MBS, and LTR (Supplementary Table 8 and Figure S6C). Likewise, the expression of *AtMYB21* and *AtMYB24* was rapidly induced under MeJA (Stracke et al., 2001). In *B. distachyon*, the expression of *BdMYB78*/*BdMYB89* and *Oryza sativa MYB91* was upregulated by ethylene, MeJA, and ABA treatment (Zhu et al., 2015; Chen S. et al., 2017). Similarly, in *Tamarix hispida*, the expression of the majority of *MYB* genes was significantly upregulated under ABA and MeJA hormone treatments (Zhang T. et al., 2018). It was observed that the total floral volatile contents were significantly increased under these hormone treatments. The expression level of *HcMYB75/79/145/238* was significantly induced under auxin, MeJA, and ABA treatments, while *HcMYB248* was significantly downregulated (Figure 4). Similarly, the expression of *OsMYB511* and *CMYB1* was induced by ABA treatment (Duan et al., 2014; Huang et al., 2015). Moreover, the expression of *HcMYB79*/*145* was dramatically induced, while *HcMYB7/8/248* was significantly downregulated under ethylene treatment. Our findings support the fact that MYB transcription factors regulate the floral aroma via controlling the key structural genes. The data indicate that under hormone treatments, the expression of *HcMYB* genes change, and the amount of floral volatile increased via upregulating the expression of key structural volatile biosynthesis genes, implying that the process of floral scent formation is associated with hormone signal transduction.

## Conclusion

In this study, a total of 253 *HcMYB* genes were identified from *H. coronarium* genome. Gene structure analysis, chromosomal location, evolutionary relationship, and expression pattern were comprehensively analyzed. Moreover, functional characterization of important candidates *HcMYB* genes revealed that they play a key role in the biosynthesis and emission of floral aroma. Furthermore, subcellular localization and protein interaction analysis revealed that candidate *HcMYB* genes transactivate the promoters of key structural volatile synthesis genes. Overall, our findings will facilitate further investigation related to floral scent formation, which is very important for aromatic plant breeding.

## Data Availability Statement

All datasets generated for this study are included in the article/Supplementary Material and in Zenodo database 10.5281/zenodo.4387064 (Repository link), further inquiries can be directed to the corresponding author/s.

## Author Contributions

FA, YF, and RY conceived and designed the concept and revised and finalized the manuscript. FA, YK, and YZ performed the experiments. FA, MW, YZ, and UA analyzed the data. CW, XW, YyY, XL, and YcY did the formal analysis. FA and YK drafted the manuscript. All authors endorsed the final version of the manuscript.

## Conflict of Interest

The authors declare that the research was conducted in the absence of any commercial or financial relationships that could be construed as a potential conflict of interest.

## Footnotes

1 https://www.Arabidopsis.org/

2 http://www.solgenomics.net

3 http://smart.emblheidelberg.de/

4 http://cn.expasy.org/tools/protparam.html

5 http://wolfpsort.seq.cbrc.jp/

6 http://gsds.cbi.pku.edu.cn/

7 http://meme-suite.org/

8 http://weblogo.berkeley.edu/logo.cgi

9 http://bioinformatics.psb.ugent.be/webtools/plantcare/html/

10 https://www.dna.affrc.go.jp/PLACE/?action=newplace

## Abbreviations

AbA: Aureobasidin A
ABA: Abscisic acid
Aux/IAA: Auxin/Indole-3-Acetic Acid
BSMT: Salicylic acid/benzoic acid methyltransferase
cDNA: Complementary DNA
GC-MS: Gas chromatography-mass spectrometer
GFP: Green fluorescent protein
NLS: Nuclear localization signal
TPS: Terpene synthase
DBD: DNA-binding domain
ERE: Ethylene responsive elements
GO: Gene Ontology
JA: Jasmonic acid
MeJA: methyl jasmonate
MEME: Multiple Em for Motif Elicitation
NJ: Neighbor-Joining
ORF: Open reading frame
PlantTFDB: Plant Transcription Factor Database
RNA-seq: RNA-sequencing
SA: Salicylic acid
SMART: Simple Modular Architecture Research Tool
TAIR: The Arabidopsis Information Resource
ABRE: ABA-responsive element
CDS: coding domain sequence
Ka: non-synonymous substitution rate
Ks: synonymous substitution rate
Mya: million years ago
qRT-PCR: quantitative real-time PCR.
