# Supplementary material for: Genome-Wide Analysis Reveals the Potential Role of MYB Transcription Factors in Floral Scent Formation in Hedychium coronarium
Source: Front Plant Sci. 2021 Feb 26;12:623742. doi: 10.3389/fpls.2021.623742 (PMC7952619; doi:10.3389/fpls.2021.623742)
Supplement: Supplementary file 2 [file Data_Sheet_1.PDF]

Genome-wide analysis reveals the potential role of MYB transcription factors in floral scent formation in *Hedychium coronarium*

Farhat Abbas[1,†], Yanguo Ke[1,3†], Yiwei Zhou[1], Yunyi Yu[1], Muhammad Waseem[4], Umair Ashraf[5], Chutian Wang[1], Xiaoyu Wang[1], Xinyue Li[1], Yuechong Yue[1], Rangcai Yu[2], Yanping Fan[1,6]*

[1]The Research Center for Ornamental Plants, College of Forestry and Landscape Architecture, South China Agricultural University, Guangzhou 510642, China
[2]College of Life Sciences, South China Agricultural University, Guangzhou 510642, China
[3]College of Economics and Management, Kunming university, Kunming 650214, China
[4]State Key Laboratory for Conservation and Utilization of Subtropical Agro-Bioresources, College of Horticulture, South China Agricultural University, Guangzhou 510642, China
[5]Department of Botany, Division of Science and Technology, University of Education, Lahore, 54770, Punjab, Pakistan
[6]Guangdong Key Laboratory for Innovative Development and Utilization of Forest Plant Germplasm, South China Agricultural University, Guangzhou 510642, China

*Corresponding email: fanyanping@scau.edu.cn
†These authors contribute equally

Supplementary data

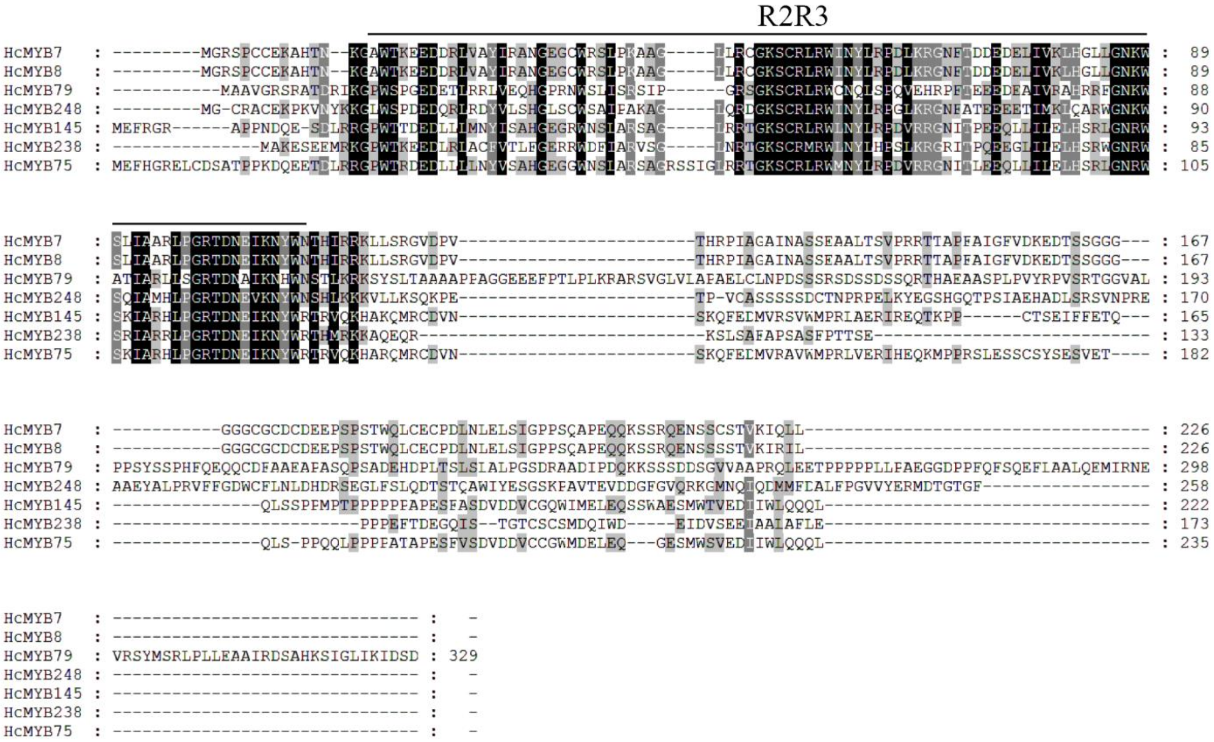

Supplementary Figure 1: Multiple sequences alignment of candidate *HcMYB* genes.

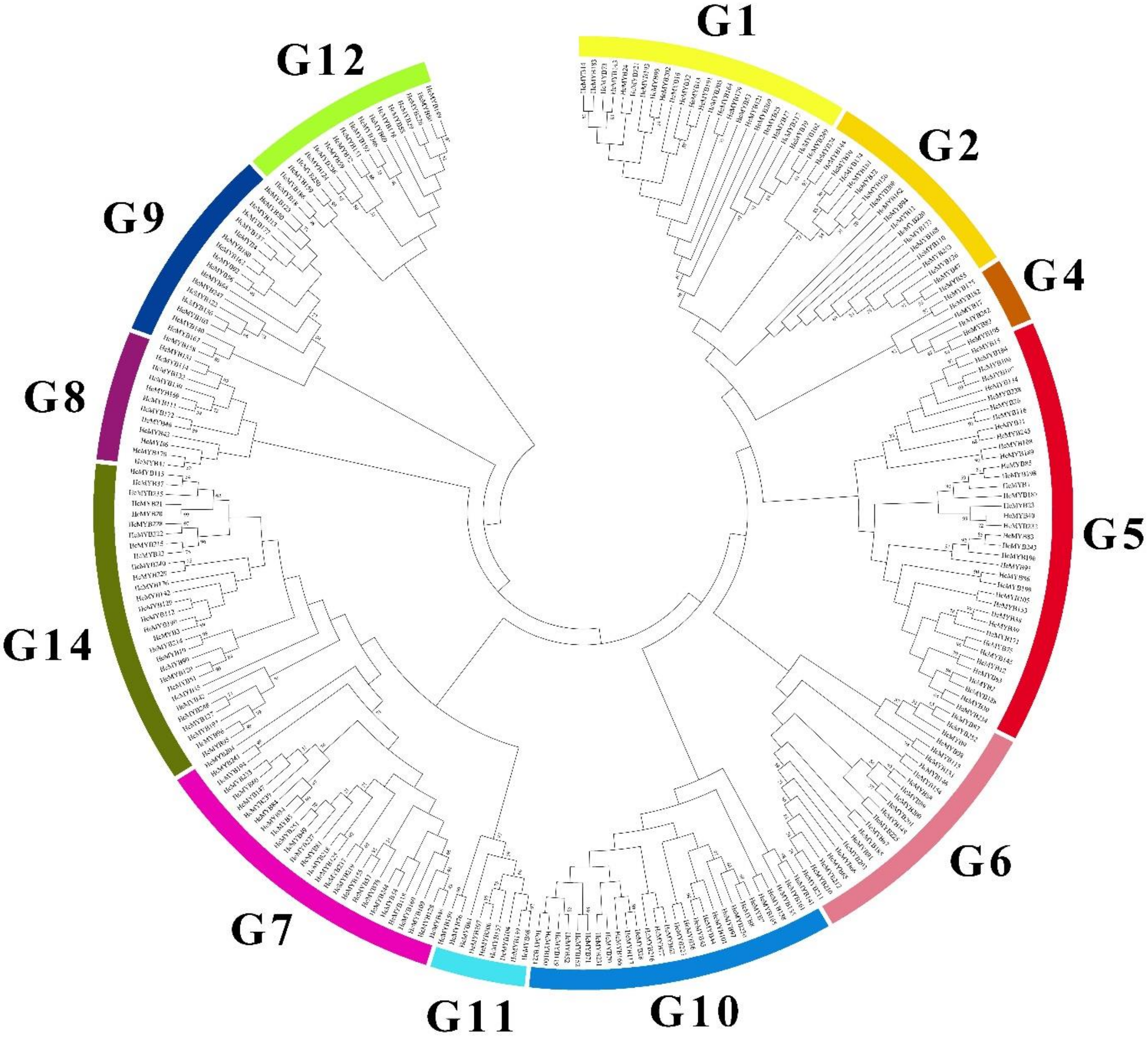

Supplementary Figure 2: Phylogenetic analysis of 253 HcMYB proteins.

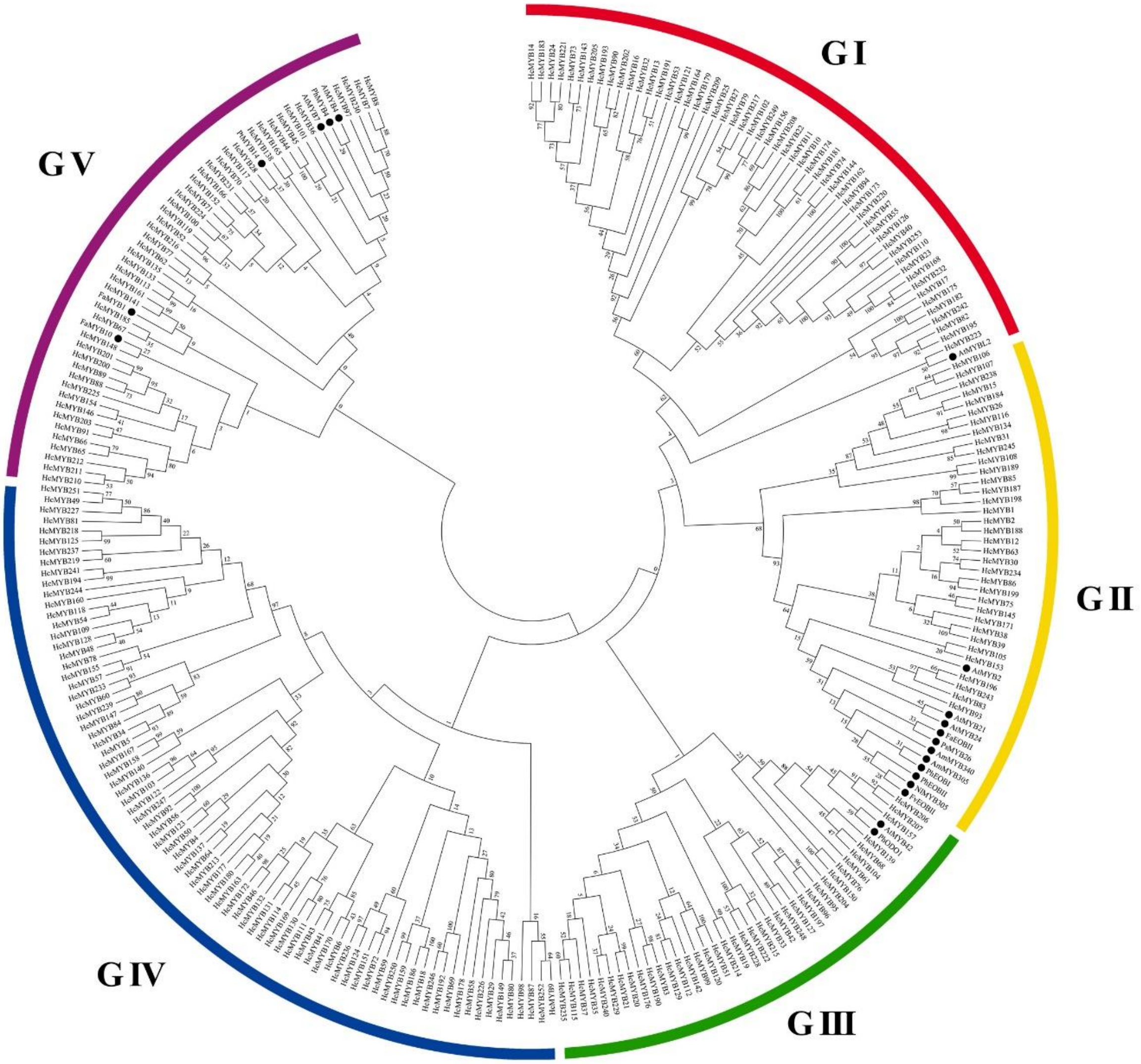

Supplementary Figure 3: Phylogenetic analysis of 253 HcMYB proteins with previously known scent-related MYBs.

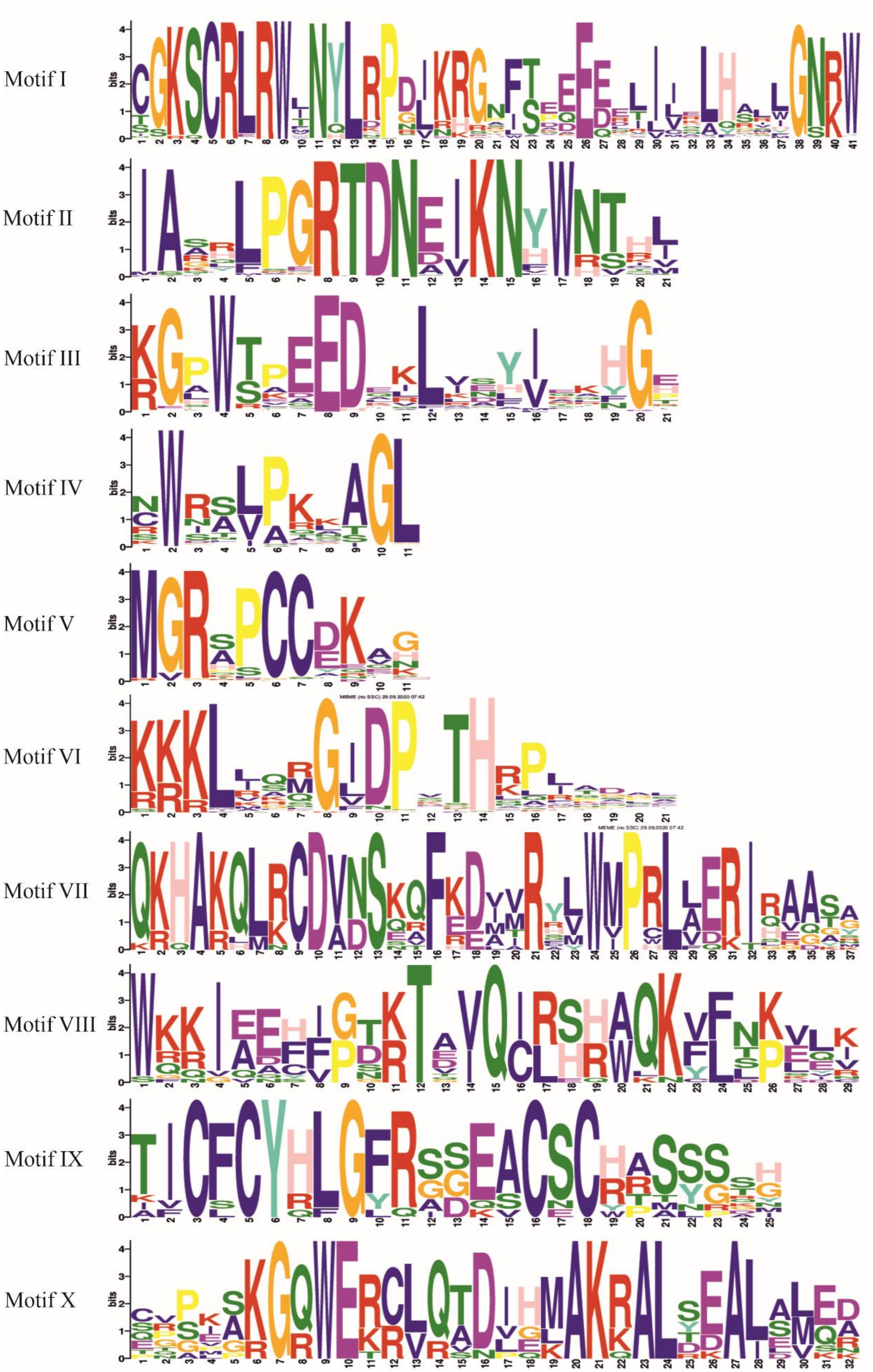

Supplementary Figure 4: The schematic representation of the individual motifs.

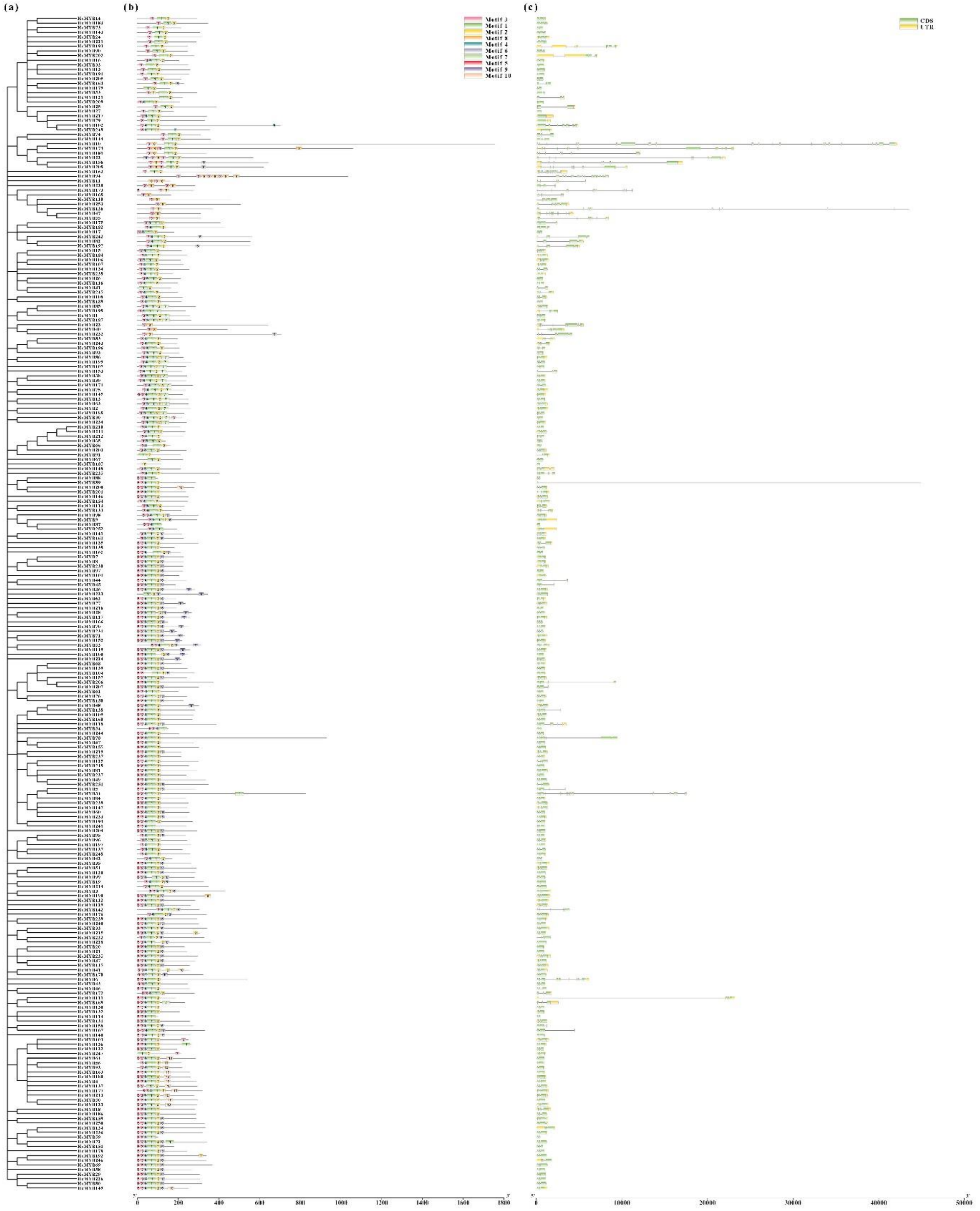

Supplementary Figure 5: Phylogenetic relationships, gene structure, and motif composition of HcMYBs

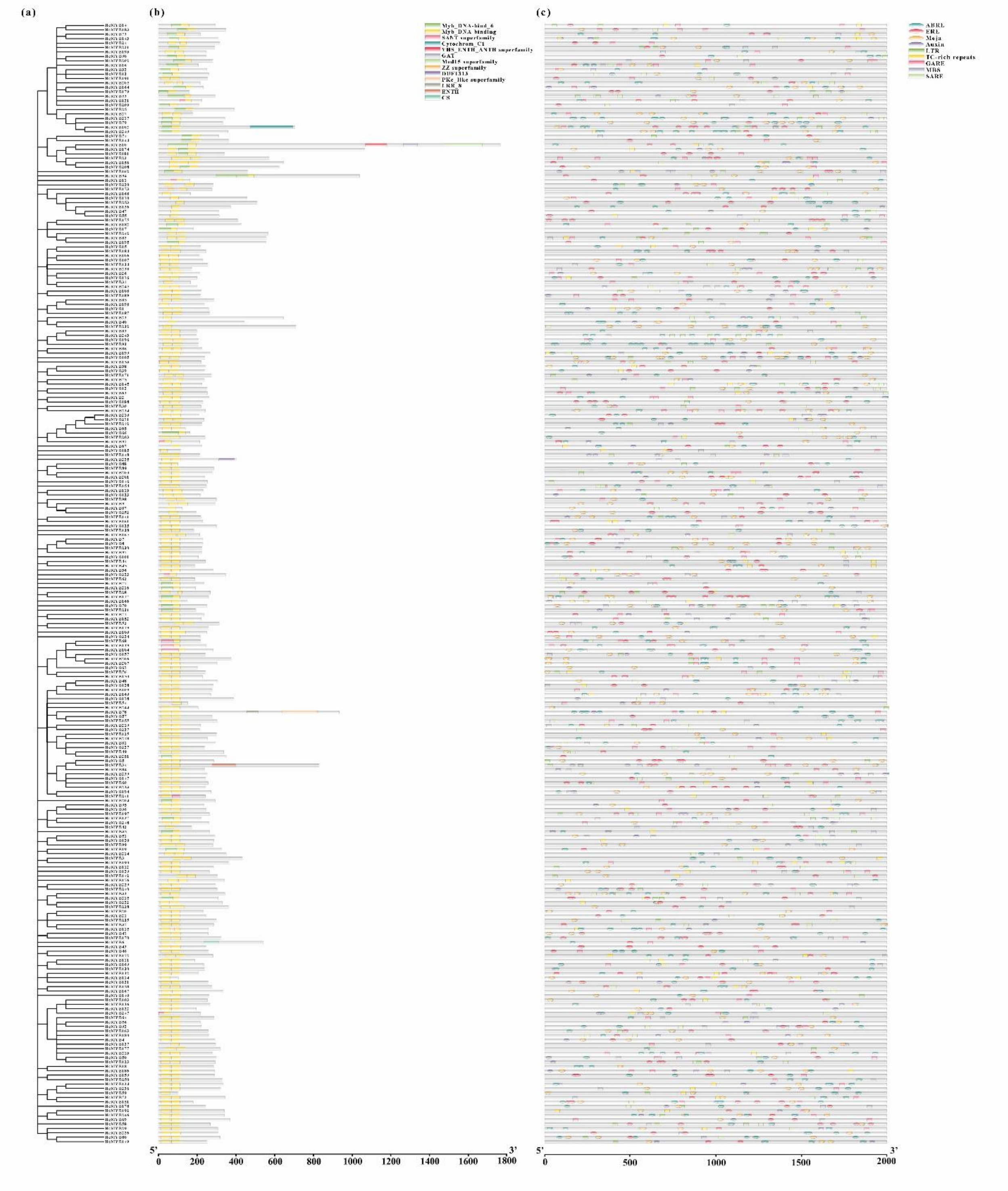

Supplementary Figure 6: Phylogenetic relationships (a), domain analysis (b), and (c) *cis*-regulatory elements in the promoters of 253 *HcMYB* genes.

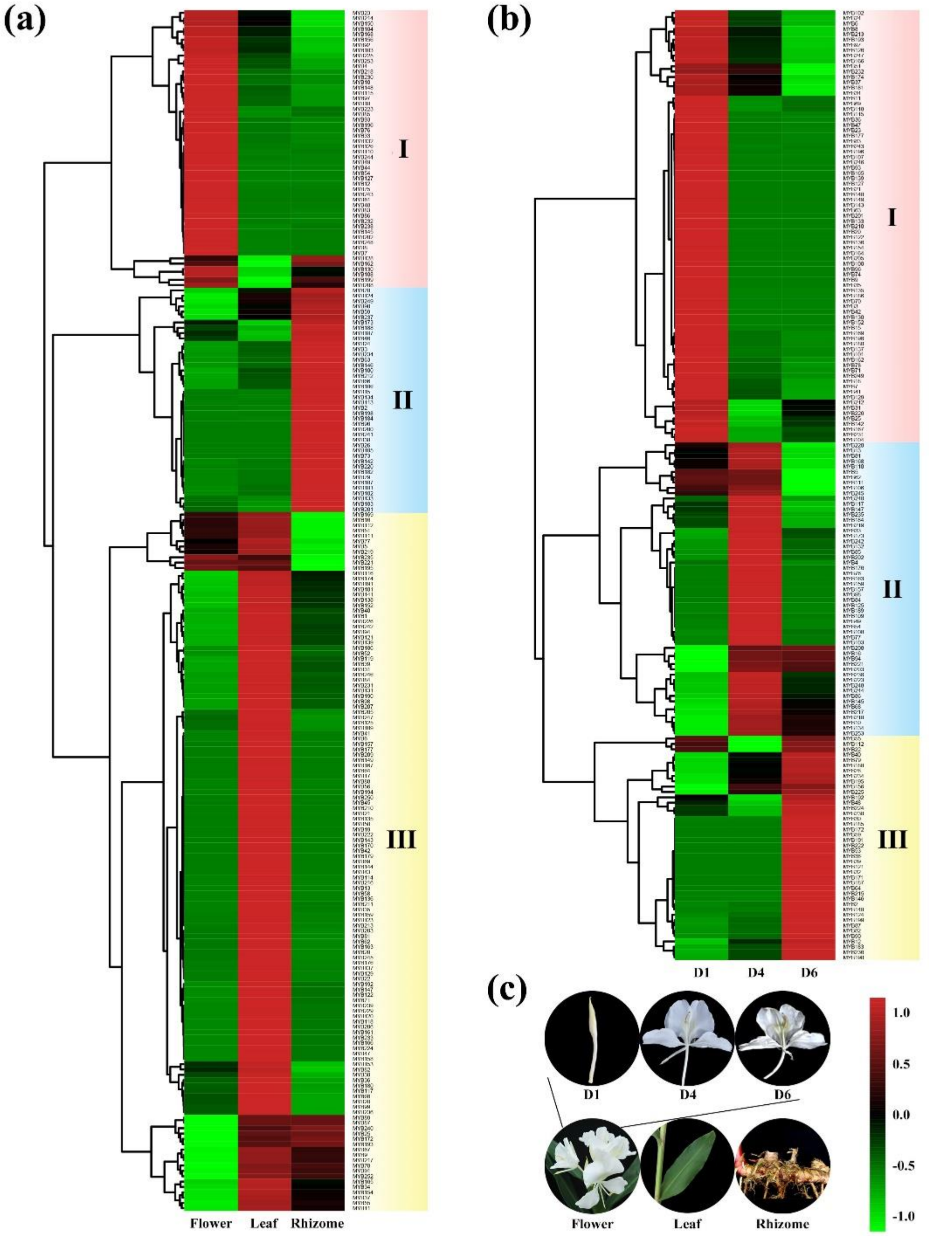

Supplementary Figure 7: Tissue-specific expression profiles of *HcMYB* genes.

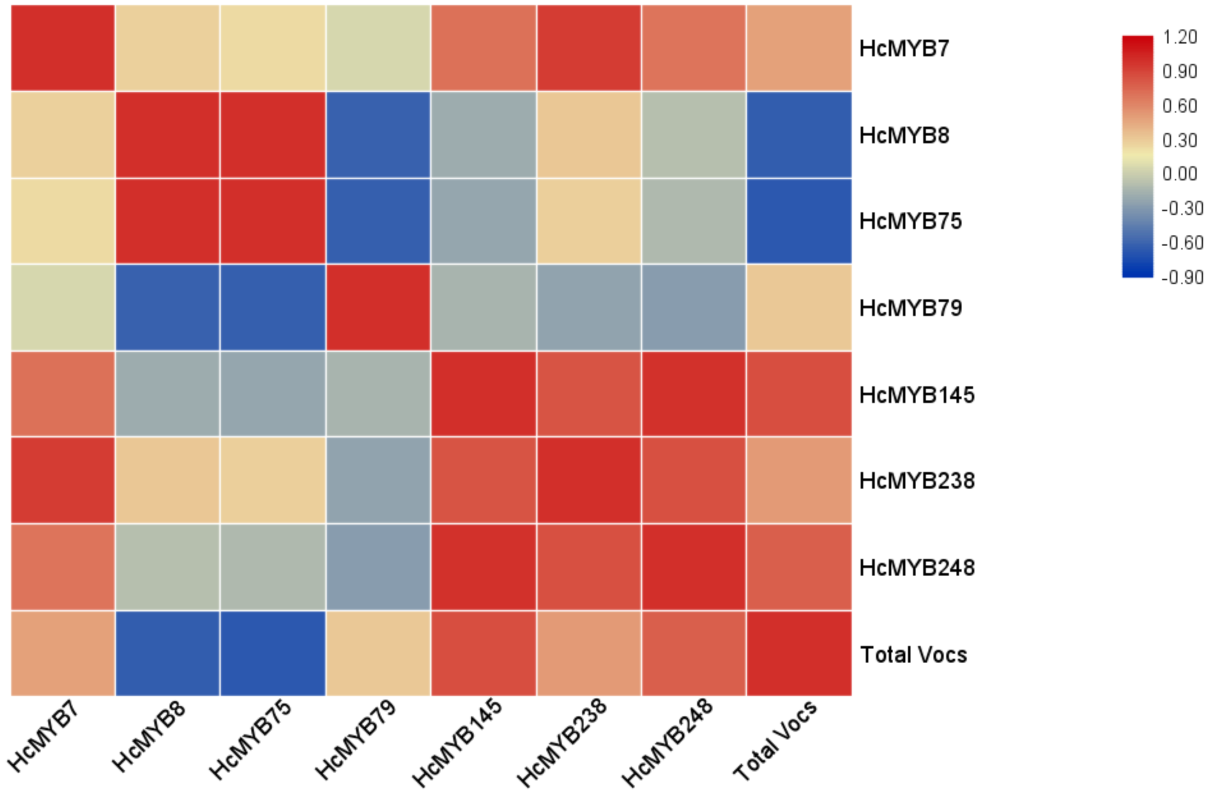

Supplementary Figure 8: The correlation analysis between the candidate *HcMYB* gene expression and total volatile compounds.

Supplementary Tables

Supplementary Table 1: Detailed characteristics of *HcMYB* genes in *H. coronarium*.

Supplementary Table 2: Primers used in the experiments.

Supplementary Table 3: The Ka/Ks ratios and estimated divergence time for segmentally and tandemly duplicated *HcMYB* genes.

Supplementary Table 4: The Ka/Ks ratios and estimated divergence time for orthologous HcMYB proteins between *H. coronarium* and *Arabidopsis thaliana*.

Supplementary Table 5: The Ka/Ks ratios and estimated divergence time for orthologous HcMYB proteins between *H. coronarium* and *Oryza sativa*.

Supplementary Table 6: The Ka/Ks ratios and estimated divergence time for orthologous HcMYB proteins between *H. coronarium* and *Ananas comosus*.

Supplementary Table 7: The Ka/Ks ratios and estimated divergence time for orthologous HcMYB proteins between *H. coronarium* and *Musa acuminata.*

Supplementary Table 8: Numbers of known stress-related elements in the promoter regions of *HcMYB* genes

Supplementary Table 9: List of *HcMYB* genes targeted by miRNAs were identified using psRNATarget.

Supplementary Table 10: MYB-core motifs in the promoters of bottom structural volatile biosynthesis genes.

Supplementary Table 11: Amino acid sequences of 253 HcMYBs

Supplementary Table 12: Coding sequences of 253 *HcMYBs*
